# Evolutionary Model of Cluster Divergence of the Emergent Marine Pathogen *Vibrio vulnificus*: From Genotype to Ecotype

**DOI:** 10.1128/mBio.02852-18

**Published:** 2019-02-19

**Authors:** Mario López-Pérez, Jane M. Jayakumar, Jose M. Haro-Moreno, Asier Zaragoza-Solas, Geethika Reddi, Francisco Rodriguez-Valera, Orr H. Shapiro, Munirul Alam, Salvador Almagro-Moreno

**Affiliations:** a Burnett School of Biomedical Sciences, College of Medicine, University of Central Florida, Orlando, Florida, USA; b Evolutionary Genomics Group, División de Microbiología, Universidad Miguel Hernández, Alicante, Spain; c Institute for Postharvest and Food Sciences, Volcani Research Center, Rishon LeZion, Israel; d Molecular Ecology and Metagenomics Laboratory, International Center for Diarrheal Disease Research, Dhaka, Bangladesh; e National Center for Integrated Coastal Research, University of Central Florida, Orlando, Florida, USA; Massachusetts Institute of Technology; University of California, San Diego; University of Southern California

**Keywords:** *Vibrio vulnificus*, emergence, evolution, genomics, waterborne pathogens

## Abstract

Vibrio vulnificus, an opportunistic pathogen, is the causative agent of a life-threatening septicemia and a rising problem for aquaculture worldwide. The genetic factors that differentiate its clinical and environmental strains remain enigmatic. Furthermore, clinical strains have emerged from every clade of V. vulnificus. In this work, we investigated the underlying genomic properties and population dynamics of the V. vulnificus species from an evolutionary and ecological point of view. Genome comparisons and bioinformatic analyses of 113 V. vulnificus isolates indicate that the population of V. vulnificus is made up of four different clusters. We found evidence that recombination and gene flow between the two largest clusters (cluster 1 [C1] and C2) have drastically decreased to the point where they are diverging independently. Pangenome and phenotypic analyses showed two markedly different lifestyles for these two clusters, indicating commensal (C2) and bloomer (C1) ecotypes, with differences in carbohydrate utilization, defense systems, and chemotaxis, among other characteristics. Nonetheless, we identified frequent intra- and interspecies exchange of mobile genetic elements (e.g., antibiotic resistance plasmids, novel “chromids,” or two different and concurrent type VI secretion systems) that provide high levels of genetic diversity in the population. Surprisingly, we identified strains from both clusters in the mucosa of aquaculture species, indicating that manmade niches are bringing strains from the two clusters together. We propose an evolutionary model of V. vulnificus that could be broadly applicable to other pathogenic vibrios and facultative bacterial pathogens to pursue strategies to prevent their infections and emergence.

## Abstract (precis)

Vibrio vulnificus is an emergent marine pathogen and is the cause of a deadly septicemia. However, the genetic factors that differentiate its clinical and environmental strains and its several biotypes remain mostly enigmatic. In this work, we investigated the underlying genomic properties and population dynamics of the V. vulnificus species to elucidate the traits that make these strains emerge as a human pathogen. The acquisition of different ecological determinants could have allowed the development of highly divergent clusters with different lifestyles within the same environment. However, we identified strains from both clusters in the mucosa of aquaculture species, indicating that manmade niches are bringing strains from the two clusters together, posing a potential risk of recombination and of emergence of novel variants. We propose a new evolutionary model that provides a perspective that could be broadly applicable to other pathogenic vibrios and facultative bacterial pathogens to pursue strategies to prevent their infections.

## INTRODUCTION

The family *Vibrionaceae* encompasses a ubiquitous group of moderately halophilic bacteria that are natural inhabitants of marine and brackish environments (1). Over the past decades, the number of *Vibrio*-related human infections rose steadily, with a similar increase observed in *Vibrio* infections in aquaculture environments (2–5). This rise in *Vibrio* virulence and pathogenicity is often attributed to the ongoing increase in sea surface temperatures associated with climate change (6, 7). Indeed, the distribution and geographical range of these opportunistic pathogens has been gradually widening, with outbreaks of *Vibrio* infections reported at latitudes as high as the Baltic Sea (4) or Alaska (8), previously considered too cold for *Vibrio* to thrive.

While the majority of the more than 100 described *Vibrio* species are harmless to humans, several species have emerged as opportunistic human pathogens, most notably Vibrio cholerae, V. parahaemolyticus, and V. vulnificus (9, 10). *Vibrio* infections are associated with a wide range of diseases and symptoms ranging from cholera, and other gastrointestinal infections, to necrotizing fasciitis and acute septicemia (1). *Vibrio* infections occur through the consumption of contaminated water or of raw or undercooked seafood or through exposure of open wounds to seawater (11, 12). According to CDC reports, an estimated 80,000 illnesses, 500 hospitalizations, and 100 deaths in the United States occur annually due to *Vibrio* infections (13). The increase in prevalence of *Vibrio* infections in the United States is unique among foodborne pathogens, with all infections associated with other major pathogens such as *Salmonella*, Escherichia coli, *Campylobacter*, *Listeria*, or *Shigella* steadily decreasing over the same time period (14).

Among vibrios, V. vulnificus has gained particular notoriety as an opportunistic and often fatal human pathogen and as an emergent pathogen of aquaculture species (12, 15). Specifically, V. vulnificus infects humans through consumption of raw seafood, causing severe gastroenteritis, or by direct contact of an open wound with seawater, producing wound infections, leading to necrotizing fasciitis or primary septicemia (16, 17). V. vulnificus is responsible for up to 94% of noncholera *Vibrio*-related deaths (12). Most deaths occur in patients with preexisting conditions, such as a compromised immune system or elevated serum iron levels (primarily in alcohol-associated liver cirrhosis), where primary septicemia may lead to mortality rates of over 50% (18, 19).

Strains of V. vulnificus are currently subdivided into three biotypes based on their biochemical characteristics and phylogeny (20, 21). Biotype 1 is associated with most of the clinical infections. Biotype 2 is primarily considered a pathogen of aquaculture-raised species, particularly eels, but is also found in association with human infections (22). Biotype 3, the smallest and most recently discovered, is thus far limited to Israel, where it caused a serious outbreak of wound infections among fish farmers and consumers of tilapia grown in aquaculture (12). A recent classification based on phylogenetic lineages broadly matches the biotype classification (23).

In contrast to V. cholerae, where all strains capable of causing cholera belong to a single clade, a comparison between V. vulnificus strains reveals a more complex pattern in the distribution of its clinical strains (24–27). Clinical V. vulnificus strains encompass a large range of genomic diversity (12, 28, 29) and lack specific markers that can be used to clearly distinguish isolates with pathogenic potential (16). Despite possessing a wide range of essential virulence factors (e.g., capsular polysaccharide [CPS], iron acquisition, cytotoxicity systems, etc.), the general consensus is that virulence in V. vulnificus is mostly host dependent (12, 16, 30). Nonetheless, it remains puzzling why most of the clinical strains isolated to date belong to biotype 1.

In this study, we aimed to gain insights into the emergence of this enigmatic human pathogen by understanding the evolutionary differences at the population, genomic, and phenotypic levels that differentiate strains belonging to the different V. vulnificus biotypes. To this end, we performed the most comprehensive computational analysis of V. vulnificus to date. For our analysis, we compared all V. vulnificus genomes currently available in public databases, comprising a total of 113 worldwide isolates from various habitats and hosts collected over a period of 40 years. We analyzed these genomes using a diverse suite of bioinformatic tools and performed phenotypic analyses of representative strains in order to infer the mechanistic processes driving their evolution. We identified four major clusters: cluster 1 (C1) to cluster 4 (C4). We show that the two largest and most divergent ones (C1 and C2) are adapted to different lifestyles that may include behavioral barriers leading to speciation. Nonetheless, frequent exchange of mobile genetic elements (MGEs) across family barriers occurs. Surprisingly, we identified strains from both C1 and C2 cohabitating in the mucosa of eels from aquaculture farms, which raises the concern of manmade environments bringing strains of these two clusters together. Overall, our findings shed light on the underlying genomic properties that are required for the emergence of pathogenic V. vulnificus strains and determine their host range. Information derived from our results may be applied to develop novel strategies for the prevention of future infections in aquaculture environments and subsequent spread to human hosts.

## RESULTS

### Phylogenomic and population structure of V. vulnificus.

**FIG 1:**
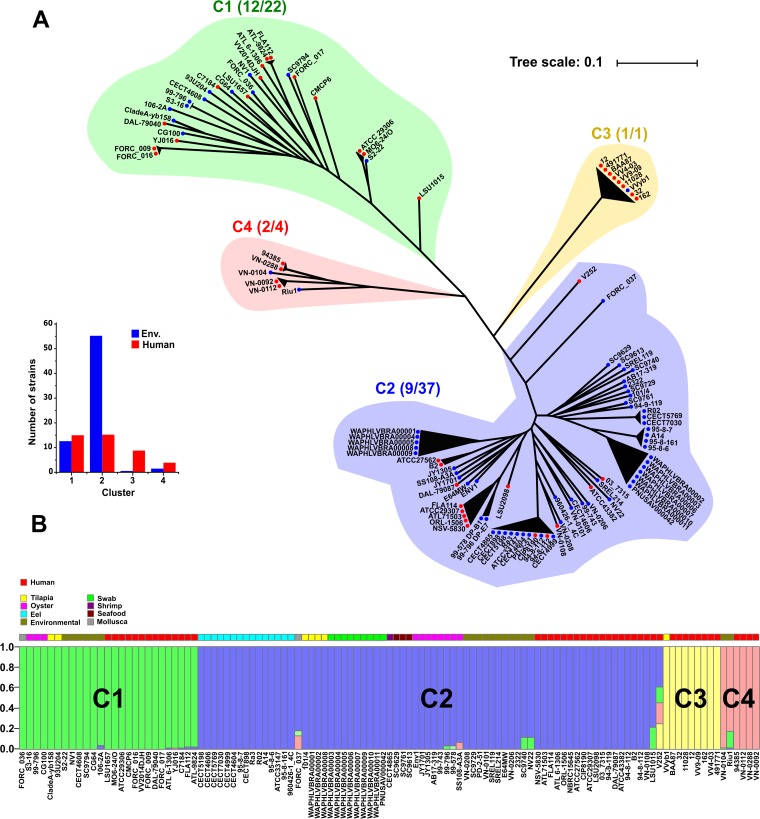
Phylogenomic and population structure of V. vulnificus. (A) Maximum likelihood tree reconstructed from single nucleotide polymorphisms of the core genome. Blue and red circles mark strains isolated from environmental and human samples, respectively. Members of the same cluster (C1 to C4) (ANI > 97%) are indicated with the same color. The smaller inset shows the proportions of nonclonal human and environmental isolates in each cluster. (B) STRUCTURE plot showing contribution to each strain from each of four ancestral populations (colored). Each vertical line represents one of the V. vulnificus strains. The color chart at the top of the plot indicates the isolated source of the corresponding strains.

In order to investigate the evolutionary changes that led to the divergent expansion of V. vulnificus, we compared 113 publicly available genomes in GenBank, complete or draft, using a phylogenomic tree based on both single nucleotide polymorphisms (SNPs) and average nucleotide identity (ANI). A total of 27,366 SNPs were identified among the aligned core genomes of 113 strains recovered from a wide range of geographical and ecological sources (see Table S1 in the supplemental material) to produce a phylogenomic tree. Using both approximations, all strains were clustered into four groups with ANI values of >97% (Fig. 1A; see also Fig. S1 in the supplemental material), here referred to as clusters 1 to 4 (C1 to C4) for simplicity.

10.1128/mBio.02852-18.1 FIG S1 Heat map showing average nucleotide identity (ANI) data determined by pairwise comparison of the genomes. The upper and left panels show the consensus hierarchy plot corresponding to the average ANI values. Circles indicate the origin of the isolates (red, human; blue, environment). The bottom and right panels show the name of each strains and the classifications into the four clusters (C1 to C4). Download FIG S1, PDF file, 0.1 MB. Copyright © 2019 López-Pérez et al. 2019 López-Pérez et al. This content is distributed under the terms of the Creative Commons Attribution 4.0 International license.

10.1128/mBio.02852-18.10 TABLE S1 Vibrio vulnificus genomes analyzed in this study. Year, host, and place of isolation are indicated. Download Table S1, XLSX file, 0.03 MB. Copyright © 2019 López-Pérez et al. 2019 López-Pérez et al. This content is distributed under the terms of the Creative Commons Attribution 4.0 International license.

The genetic population structure, inferred based on the pattern of SNPs shared among the strains (Fig. 1B), revealed four ancestral populations that closely corresponded with the phylogenomic approach. We did not observe significant mixing between the different clusters, suggesting low connectivity and gene flow. Significantly, our analysis indicated that clusters 1 and 2 are widely divergent lineages (ANI, ca. 95%) and are on the verge of qualifying as different species (Fig. S1). For our subsequent analyses, we focused on clusters C1 and C2, which between them include close to 90% of the strains, including the bulk of the diversity of clinical isolates. Indeed, the low number of representatives in cluster 3 (C3) and cluster 4 (C4), combined with their high clonality, did not provide enough genetic diversity to obtain meaningful results. Nonetheless, future studies should analyze additional strains from C3 and C4, with higher divergence, to obtain a more comprehensive view of the evolution and emergence of additional V. vulnificus clusters.

Despite the genomic divergence among clusters, we could not identify a distinct pattern linking strain phylogeny, source of isolation, and virulent capabilities (Fig. 1). Nevertheless, C1 appears to contain a significantly higher proportion of strains isolated from humans, while C2 is dominated by strains derived from multiple marine hosts, including a large proportion of eels, and appears to be closely associated with aquaculture environments (Fig. 1B). An additional distinguishing feature is the relatively high clonality of C2 strains, both clinical and environmental, compared to a much higher divergence among strains belonging to C1 (Fig. 1A). Combined, these observations point toward different evolutionary pathways taken by these clusters that may be partially driven by anthropogenic influences.

### Evolution and genomic diversity of V. vulnificus clusters.

In order to further elucidate the evolutionary drivers behind the divergence of the two main clusters, we looked for signatures of genetic drift acting on the genome of each cluster. Assembly of 12 clinical and environmental strains from each cluster showed that the synteny in both chromosomes was remarkably well preserved within and also between clusters, including positions of the main features of the flexible genome (e.g., CPS and the superintegron) (Fig. S2). The overall means of the estimates of the averages of nonsynonymous (d*N*) to synonymous (d*S*) substitution rates for the analyzed genomes were 0.42 ± 0.02 and 0.46 ± 0.01 for C1 and C2, respectively, indicating weak stabilizing selection on both clusters. Similar dN/dS values were obtained for bacterial pathogens such as Chlamydia trachomatis (0.40) (31), Salmonella enterica serovar Typhi (0.45) (32) and Burkholderia mallei (0.47) (33).

10.1128/mBio.02852-18.2 FIG S2 Genome comparison of several genomes from environmental and human origins for the two clusters. The genomic islands identified have been highlighted in purple and green for C1 and C2, respectively, and the inferred function is indicated on the top panel. Download FIG S2, PDF file, 0.07 MB. Copyright © 2019 López-Pérez et al. 2019 López-Pérez et al. This content is distributed under the terms of the Creative Commons Attribution 4.0 International license.

We found substantial differences in the degree of divergence of the genomes within each cluster. Similarly to other *Vibrio* species, V. vulnificus possess two chromosomes with different sizes. The size of the large chromosome is ca. 3.2 Mb, whereas the size of the small chromosome is ca. 1.8 Mb. Despite having a lower relative abundance of strains, both chromosomes of C1 were found to be genetically more diverse than those of C2 and to be accumulating greater amounts of SNPs (Fig. S3). Furthermore, pairwise nucleotide diversity was higher in both clusters in chromosome 2 (Chr2) (Fig. S3), showing that genes are evolving faster in that chromosome than in chromosome 1 (Chr1). To further assess the relative effects of recombination and mutation between the two chromosomes of strains belonging to the two main clusters, we estimated the ratio of recombination events to point mutation events (R/θ) (34). The mean R/θ values for all strains from both clusters were 0.38 ± 0.02 for Chr1 and 0.41 ± 0.01 for Chr2. However, for strains within C1, ratios of recombination-associated replacements were higher in Chr I (0.69 ± 0.002) even though the R/θ values were similar for the second chromosome (0.32 ± 0.001). Similar R/θ values (0.85 ± 0.008 for Chr I and 0.30 ± 0.001 for Chr II) were estimated within C2 representatives.

10.1128/mBio.02852-18.3 FIG S3 Pairwise comparisons among the members within the two clusters to analyze the average number of SNPs per kilobase normalized by the core genome of the corresponding chromosome. Download FIG S3, PDF file, 0.02 MB. Copyright © 2019 López-Pérez et al. 2019 López-Pérez et al. This content is distributed under the terms of the Creative Commons Attribution 4.0 International license.

These data indicate low gene flow between the clusters and limited recombination between them, possibly leading toward speciation (Fig. 1). The most plausible scenario that explains our findings is that physical isolation has decreased the probability of encounter and recombination between the two clusters leading to allopatric speciation and the generation of distinct ecotypes. This model of bacterial ecotype evolution and separation restricting recombination has already been observed previously in pathogens such as Yersinia enterocolitica (35) and Campylobacter jejuni (36) and in populations of the hyperthermophilic archaeon Sulfolobus (37).

### Virulence factors and capsular polysaccharide diversity.

Next, we compared data corresponding to the presence and distribution of known virulence factors in both clusters. In order to investigate this, we contrasted all protein-coding genes from the V. vulnificus strains against the Virulence Factors Database (38). We considered putative virulence factors for all genes sharing over 90% amino acid sequence identity with any entry in that database (Fig. S4). All V. vulnificus strains possess a wide array of putative virulence factors related to attachment and adhesion, iron acquisition, quorum sensing, secretion, and cytotoxicity systems. Surprisingly, with the exception of the CPS cluster, the virulence factors were similarly distributed in all strains regardless of cluster, source of isolation, or clinical/environmental designation (Fig. S4). The prevalence of antibiotic resistance genes, analyzed using the MegaRES database (39), pointed to an intrinsic resistance to tetracycline, with *tet*(34) and *tet*(35) genes present in the core genome of all the strains.

10.1128/mBio.02852-18.4 FIG S4 Phylogenetic structure and distribution of key virulence factors within V. vulnificus. The maximum likelihood tree was reconstructed from single nucleotide polymorphisms of the core genome. The branches are colored in accordance with the four clusters (C1 to C4). Presence (black squares) or absence (blank space) as determined by analysis using the VFDB virulence factor database (>90% identity [ID]) is shown for all the strains. Download FIG S4, PDF file, 4.0 MB. Copyright © 2019 López-Pérez et al. 2019 López-Pérez et al. This content is distributed under the terms of the Creative Commons Attribution 4.0 International license.

**FIG 2:**
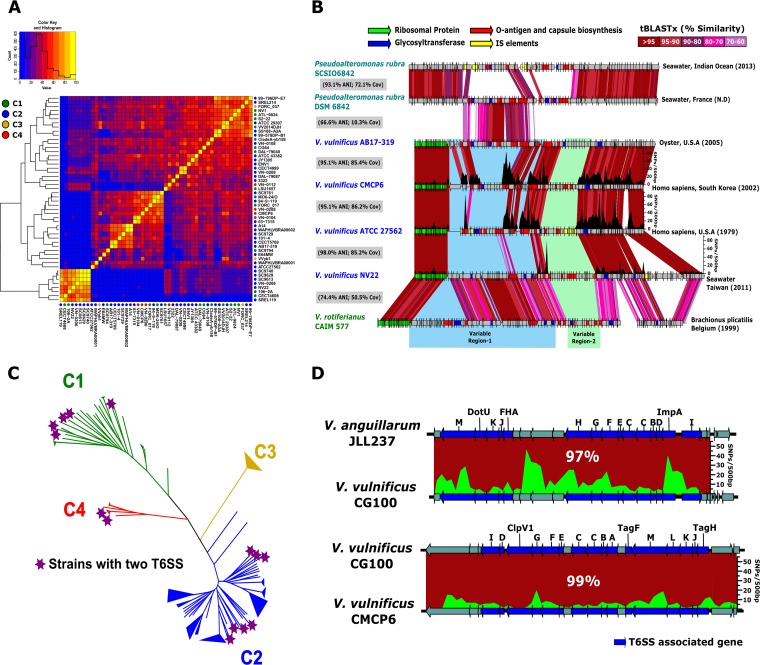
Capsular polysaccharide and type VI secretions system genomic region comparison. (A) Hierarchical clustering of pairwise average nucleotide identity comparison of the variable region of the capsular polysaccharide genomic island using one representative of each clonal frame within the V. vulnificus species. The correspondence of each strain with its cluster is shown by a color-coded circle. (B) Schematic representation of the same cassettes that were found to still be syntenic but at lower similarity in members of the genus *Pseudoalteromonas*. Color-coded arrows show locations of important genomic features. The plot above the genomes indicates the number of SNPs in a 500-bp window. Variable regions 1 and 2 are highlighted in blue and green, respectively. (C) The location of the 14 strains with two type VI secretion systems (T6SS) is highlighted with a purple star in the tree reconstructed from single nucleotide polymorphisms of the core genome (Fig. 1). (D) Comparison of the two T6SS clusters found in strain CG100. Genes associated with this cluster are highlighted in blue. The plots above the genome indicate the number of SNPs in a 500-bp window.

Since the CPS, which is part of the flexible genome of V. vulnificus (Fig. S2), was found to be the most diverse virulence factor, we investigated whether its genetic diversity might yield insights into the divergence of C1 and C2. First, we extracted and generated a heat map based on data representing the similarity of the CPS genomic islands using only one representative of each clonal frame within the V. vulnificus species (Fig. 2A). Pairwise comparison showed that the variability of this region is very high, even within members of the same cluster, since we did not find two identical versions (Fig. 2A). Furthermore, genomic comparison revealed that the CPS cluster has two separate hypervariable regions (Fig. 2B). SNP analysis of the common part of the CPS showed a large number of SNPs, mostly synonymous, detected at one side of these variable regions (Fig. 2B). This phenomenon has been suggested to be the result of events of recombination between divergent genomes and might elicit the complete replacement of the gene cluster (40).

Interestingly, we identified several syntenic groups in distantly related genomes. Highly similar CPS clusters (ca. 70% to 80% tBLASTx identity) were found in comparisons between V. vulnificus NV22 and Vibrio rotiferianus CAIM 577 (74.4% ANI, 50.5% coverage [Cov]) (Fig. 2B), with preserved synteny and location in the genome. Similarly, the CPS cluster of V. vulnificus AB17-319 shares a syntenic cluster with Pseudoalteromonas rubra DSM6842 (66.6% ANI, 10.3% Cov), a strain isolated from seawater in Nice, France (Fig. 2B). This region contains genes that encode glycosyltransferases, aminotransferases, and polysaccharide biosynthesis proteins, among other products.

With the exception of the CPS cluster, our analyses showed virulence factors to be both widely distributed and highly conserved among all analyzed V. vulnificus strains. Here we provide evidence of two hypervariable CPS regions that are frequently exchanged by homologous recombination within and between species, as there is evidence for their import from distant taxonomic units. It is well documented that CPS is an essential virulence factor for V. vulnificus and other bacterial pathogens (19, 41–43). These highly variable regions are involved in the production of different sugar skeletons that form or decorate the extracellular structure and allow the bacterium to avoid predation by protozoa and other grazers in the natural environment (44, 45). The high variability in the CPS region of closely related microbes indicates that, even though it is essential for colonization and survival in the human host, it cannot be used as a factor to differentiate between clinical and environmental strains or between C1 and C2 strains.

### Presence of two type VI secretion systems in V. vulnificus.

The type VI secretion system (T6SS) functions as an antibacterial mechanism facilitating elimination of competing bacteria during host colonization (46–48). We hypothesized that the distribution of this key virulence factor within *Vibrio* species may thus shed light on the evolution of virulence in V. vulnificus (49). Our bioinformatic analysis revealed T6SS-associated genes in all V. vulnificus strains in our census. Moreover, these genes were invariably located in a conserved region on Chr2. Interestingly, 14 of the isolates, belonging to different clusters, harbored a second T6SS homolog (T6SS-2) on Chr2 (Fig. 2C). A phylogenetic analysis of the concatenation genes encoding the TssB and TssC proteins (Fig. S5), previously suggested as markers for evolutionary relationships between distantly related T6SS systems (50), revealed the core T6SS (T6SS-1) to be highly congruent with the phylogeny of the whole genome, suggesting vertical transmission (Fig. S5). In contrast, T6SS-2 clustered into four different groups, with similarity to other species within the genus *Vibrio* but no apparent correlation with overall phylogeny (Fig. S5). Fig. 2D shows a schematic of the two T6SS homologs from strain CG100, which encodes both systems. While T6SS-1 from this strain shared 99% similarity with the T6SS cluster from reference strain V. vulnificus CMCP6, the T6SS-2 (absent in CMCP6) showed 97% similarity to a T6SS cluster in V. anguillarum JLL237, isolated from fish tissue. To our knowledge, this is the first report of a human pathogen encoding two T6SS. It remains to be determined whether T6SS-2 contributes to the virulence of V. vulnificus. Redundancy in T6SS gene clusters has been previously described in Vibrio fluvialis (strain 85003) and Vibrio proteolyticus (51, 52). It is possible that this novel T6SS might have specificity for some bacterial or protozoal species, thus increasing the fitness of V. vulnificus in its natural environment and increasing the ability of the bacterium to outcompete the intestinal microbiota or to overcome the host’s immune system.

10.1128/mBio.02852-18.5 FIG S5 Phylogenetic tree constructed using a concatenation of TssB and TssC proteins to evaluate the diversity of the type VI secretion systems found in V. vulnificus. The branches of the tree and the name of each strain are colored in accordance with the four clusters (C1 to C4). The numbers in parentheses indicate (1) the core cluster and (2) the strains with the extra copy. Download FIG S5, PDF file, 0.05 MB. Copyright © 2019 López-Pérez et al. 2019 López-Pérez et al. This content is distributed under the terms of the Creative Commons Attribution 4.0 International license.

### V. vulnificus panmobilome.

Mobile genomic regions enable the rapid recombination of genetic elements, which may facilitate the expansion of physioecological range of a microbe, including the dissemination of antimicrobial resistance and virulence factors within a population (53–55). We analyzed the panmobilome of different V. vulnificus strains to identify unique mobile genetic elements (MGEs) associated with the different clusters.

### (i) Plasmids.

**FIG 3:**
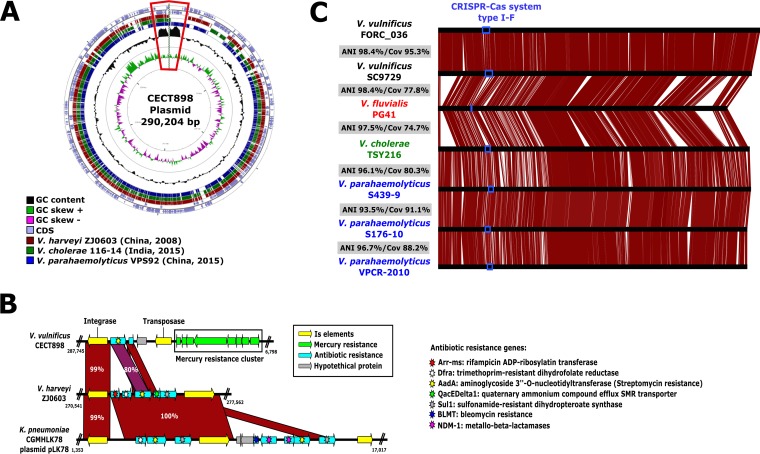
Panmobilome of V. vulnificus. (A) Circular representation of the CECT898 plasmid (290 kb). The rings are defined from outside to inside as follows: circles 1 and 2, coding DNA sequence (CDS) in the positive strand and negative strand, respectively; circles 3, 4, and 5, BLAST against Vibrio harveyi ZJ0603, Vibrio cholerae 116-14, and Vibrio parahaemolyticus VPS92; circle 6, CG content; circle 7, GC skew. The region bounded by red shows the enlarged region in section B. (B) Regions homologous to CECT898 plasmid with high GC content in Vibrio harveyi ZJ0603 and Klebsiella pneumoniae CGMHLK78 plasmid pLK78. (C) Schematic representation comparing the first two chromids described in V. vulnificus with other, similar examples found in several *Vibrio* species. CRISPR-Cas system, clustered regularly interspaced short palindromic repeat-Cas system.

Several virulence plasmids were described in V. vulnificus, primarily in strains associated with C2 (23), that were suggested to provide resistance to the innate immunity of eels (56, 57). Here we describe a novel megaplasmid (404 kb) in C1 strain V. vulnificus CECT4608, originally isolated from the water of an eel tank (Spain, 1990), that is highly similar to and syntenic with (ANI, 98.7%; Cov, 92.9%) a megaplasmid found in V. coralliilyticus strain RE98 (p380; 380 kb) isolated from a shellfish hatchery (United States, 2000) (Fig. S6A). We also identified a 290-kb conjugative plasmid in strain CECT898 (Japan, 1979) which shows significant gene and organizational similarities to plasmids found in three *Vibrio* species (Vibrio harveyi, V. cholerae, and V. parahaemolyticus) isolated over a period of 35 years from different hosts and locations in Southeastern Asia (Fig. 3A; see also Fig. S6B). An important feature of this plasmid was the presence of a region with high GC content (ca. 66% compared with ca. 43% in the rest of the plasmid) flanked by a class 1 integron-integrase gene (Fig. 3B). In close proximity to this region, we identified a complete *mer* operon conferring mercury resistance and showing high similarity to genes in distantly related microbes such as Acinetobacter baumannii, as well as to two antibiotic resistance genes, *aadA5* and *qacEdelta1*, conferring resistance to streptomycin and quaternary ammonium compounds, respectively (Fig. 3B). The presence of the integron-integrase gene, along with the 3′-conserved region containing *qacEdelta1* and *sul1* genes, clearly classifies this segment as a class 1 resistance integron, widely distributed among clinical strains involved in the capture and spread of antibiotic resistance genes (58).

10.1128/mBio.02852-18.6 FIG S6 (A) Circular representation of the CECT4608 plasmid (404 kb). The rings are defined from outside to inside as follows: circles 1 and 2, CDS in the positive strand and negative strand, respectively; circle 3, CG content; circle 4, GC skew. A schematic representation comparing plasmid CECT4608 and plasmid p380 found in V. coralliilyticus RE98 is shown at the bottom. (B) Circular representation of the CECT898 plasmid (290 kb). The rings are defined from outside to inside as follows: circles 1 and 2, CDS in the positive strand and negative strand, respectively; circle 3, CG content; circle 4, GC skew. A schematic representation comparing CECT898 plasmid and other, similar plasmids found in different *Vibrio* species is shown at the bottom. The table indicates the origin of the plasmids. Download FIG S6, PDF file, 0.7 MB. Copyright © 2019 López-Pérez et al. 2019 López-Pérez et al. This content is distributed under the terms of the Creative Commons Attribution 4.0 International license.

### (ii) V. vulnificus chromid.

Two megaplasmids (ca. 900 kb), containing all the hallmarks of a “chromid” (59), were found in V. vulnificus strains FORC_36 and SC9729, belonging to C1 and C2, respectively. Despite substantial divergence between the hosting strains (ANI, 95.7%), the chromids share markedly high similarity (ANI, 98.49%; Cov, 95.3%) (Fig. 3C). Furthermore, the GC content of these chromids is 37.2%, substantially lower than the 46.5% average GC content determined for V. vulnificus strains. Interestingly, we found five nearly identical chromids hosted by three additional *Vibrio* species (V. parahaemolyticus, V. fluvialis, and V. cholerae) (Fig. 3C). To our knowledge, this is the first evidence of the widespread distribution of this kind of conjugative element and also of its presence across the species barrier of any known microbe.

### (iii) Prophages.

We have found 77 prophage-related elements in the V. vulnificus genomes associated with all clusters, located on both chromosomes and ranging in size from 3.7 to 59 kb. The most prevalent element, found in 29 V. vulnificus genomes, was a small (ca. 12-kb) prophage encoding two toxins, similarly to V. cholerae KSF-1Φ phage (60, 61) (Fig. S7A). The phylogenomic analysis of both proteins clustered them separately into two well distinguishable branches (Fig. S7B and C). The largest protein was annotated as zonula occludens toxin (Zot), required for phage morphogenesis, which was shown in V. cholerae to increase the permeability of the intestinal epithelium (62, 63). These two proteins are also found in the lysogenic V. cholerae CTXΦ phage, although the similarities among them were lower than 30%. Furthermore, the RtsA and RtsB proteins, which are also encoded in CTXΦ and KSF-1Φ, were conserved in all the V. vulnificus sequences. In CTXΦ, RstA is required for DNA replication, whereas the RtsB gene facilitates the integration of the prophage into the V. cholerae genome (60).

10.1128/mBio.02852-18.7 FIG S7 (A) Genomic comparison of the different prophages containing enterotoxin and zonula occludens toxin within V. vulnificus strains. (B) Phylogenetic tree for the zonula occludens toxins constructed using V. cholerae CTX phage toxin as an outgroup. (C) Phylogenetic tree for the enterotoxins constructed using V. cholerae CTX phage toxin as an outgroup. Download FIG S7, PDF file, 0.09 MB. Copyright © 2019 López-Pérez et al. 2019 López-Pérez et al. This content is distributed under the terms of the Creative Commons Attribution 4.0 International license.

Overall, our findings indicate that despite divergence between C1 and C2, exchanges of MGEs appear to happen frequently and indiscriminately. Nonetheless, this phenomenon also occurs between different species, as shown by the detection of identical elements in distant relatives (Fig. 3; see also Fig. S6). Our results further highlight the importance of plasmids for the dispersion of harmful genetic determinants among groups of strains even beyond the family barriers. Furthermore, they support the idea of the role of aquatic ecosystems as antibiotic resistance reservoirs. This poses a serious problem with respect to treatment of emergent V. vulnificus strains as they have the clear potential of becoming a multidrug-resistant pathogen.

### V. vulnificus pangenome.

Given the marked divergence between the two main clusters (ANI, ca. 95%), we decided to investigate the evolutionary differences in their overall gene content through pangenome analysis. Despite the nearly 3-fold-larger number of C2 genomes, the C2 pangenome was only 12.7% larger than that of C1, possibly due to the high clonality within C2. Nevertheless, analysis of the core genome of each of the clusters indicated that C1 has a core genome that is nearly 40% larger than that of C2 (2,263 versus 1,641 genes). It seems that the core genome has already reached a plateau in both clusters (Fig. S8). The pangenome curve in both clusters has not saturated, indicating an open pangenome and high genetic diversity in V. vulnificus (Fig. S8). This interpretation is further supported by the high prevalence of “cloud genes” (e.g., genes found in up to 15% of the strains), which corresponds to approximately 70% of both pangenomes.

10.1128/mBio.02852-18.8 FIG S8 Pangenome (red) and core genome (blue) plots of the two V. vulnificus clusters analyzed. Black bars indicate the number of new genes contributed by each strain. The proportions of coding DNA sequence (CDS) in the core, soft core, shell, and cloud genome are represented in the green circle. Download FIG S8, PDF file, 0.04 MB. Copyright © 2019 López-Pérez et al. 2019 López-Pérez et al. This content is distributed under the terms of the Creative Commons Attribution 4.0 International license.

### (i) Origins of pangenome divergence.

**FIG 4:**
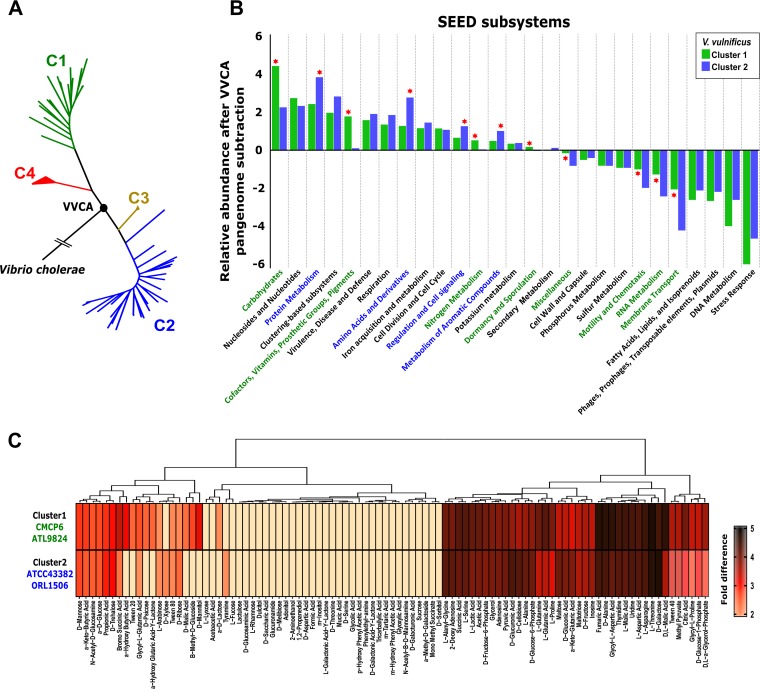
Pangenome analysis and carbon utilization of V. vulnificus strains. (A) Maximum likelihood tree reconstructed from single nucleotide polymorphisms of the core genome using V. cholerae as an outgroup. The black circle shows the root of the tree. Members of the same cluster are indicated with the same color. VVCA, Vibrio vulnificus common ancestor. (B) Functional characterization of the pangenome using the SEED Subsystems database and the difference between the two clusters and VVCA pangenome. The red stars indicate the results that differed using a cutoff criterion of fold change greater than 1.5 between the clusters. (C) Biolog phenotypic microarrays measuring bacterial ability to metabolize a variety of carbon sources by the use of PM1 phenotypic microarray plates. The heat map shows the average levels of carbon utilization of C1 representatives (CMCP6 and ATL9824) and C2 representatives (ATCC 43382 and ORL1506) in comparison to the negative control. Analyses were carried out in duplicate.

Next, we aimed to elucidate the origins of the V. vulnificus divergence by identifying a potential common ancestor node among the four clusters. Using V. cholerae as an outgroup, we determined that V. vulnificus clusters started branching out at a node close to C3 and C4 (Fig. 4A). We unified the pangenome of both C3 and C4 to generate what we termed the V. vulnificus common ancestor (VVCA). We reasoned that subtraction of VVCA from the pangenome of the highly divergent C1 and C2 clusters would provide us with a reference point and give us an unbiased and specific in-depth evolutionary history of each group at the functional level. We compared the functional classifications of the gene coding sequences from the pangenomes of C1 and C2 after subtraction of VVCA. After this process, while the core of C1 was reduced by 40% to 650 genes, the core of C2 was reduced by up to 54%, leaving the number of genes possessed in common at 1,238. Comparison against the SEED Subsystems database (64) revealed differences between the clusters in the relative abundances of genes analyzed using a cutoff criterion of fold change greater than 1.5 in comparison to their prevalence in the VVCA (Fig. 4B).

In C1, we identified an increased proportion of genes related to the functional classification “carbohydrates” (monosaccharide and aminosugars), as well as to “membrane transport,” mainly, a greater proportion of type II secretion systems, which represent the vehicle for the secretion of the degradative enzymes (e.g., proteases, nucleases, phospholipases, and chitinases) that support bacterial persistence in different environments. Interestingly, this system plays a major role in the colonization and virulence of V. cholerae through degradation of intestinal mucin and cholera toxin secretion (65, 66). We also found more genes involved in chemotaxis, within the “motility and chemotaxis” classification, the process by which motile cells modulate flagellar rotation in response to the surrounding environment (67). Finally, genes involved in “dormancy and sporulation” were significantly enriched in C1 (Fig. 4B). These genes appear to regulate entry into and resuscitation from a persister-like state called viable but nonculturable, which is essential for both pathogenesis and survival in the environment in V. cholerae and other pathogens (68, 69). “Cofactors, vitamins, prosthetic groups, and pigments” and “RNA metabolism” are two additional categories that are overrepresented in C1. Overall, differential functional characterization of C1 suggested an opportunistic (*r*-strategist or bloomer) lifestyle, typical of microbes that grow rapidly, taking advantage of the sporadic inputs of organic matter that appear in the environment.

On the other hand, genes involved in protein biosynthesis (“protein metabolism”) and synthesis of amino acids (“amino acids and derivatives”) were more abundant in C2, suggesting possible adaptation to long-term colonization of nutrient-rich environments. The skin mucus of fish and eel is rich in proteins and carbohydrates and supports diverse commensal microbial populations (70). In fact, only V. vulnificus isolates of C2 have been recovered from diseased eels cultured in brackish and freshwater farms (23). We found that systems conferring resistance against phage predation such as restriction-modification and toxin-antitoxin (“regulation and cell signaling”) were enriched in the C2 pangenome. It has been demonstrated that bacteriophages are present in higher concentrations in the mucus layers than in the surrounding environment as a defense mechanism that ultimately protects the underlying epithelium from bacterial infections (56–58). Preferential colonization of mucosal surfaces is one possible explanation for the lower level of divergence and smaller core genome of C2, as strains from this cluster might not have to encounter conditions that are as variable as those encountered by the strains that typically inhabit the water column. Finally, C2 contained an enrichment of genes involved in aromatic carbon catabolism (“metabolism of aromatic compounds”).

### (ii) Carbohydrate utilization.

The most abundant functional classification in both clusters involved genes associated with carbohydrate metabolism, with a significant overrepresentation in strains from C1 (Fig. 4B). The ability to utilize a diverse set of carbon sources has been shown to be crucial for the pathogenicity and emergence of other pathogenic vibrios (71). We further analyzed the genomic potential for carbohydrate metabolism using the Carbohydrate-Active enZYmes (CAZy) database (72). We found a significantly higher percentage of glycoside hydrolases (GHs) and polysaccharide lyases in the C1 pangenome, suggesting a greater carbohydrate degradation capacity in this cluster (Fig. S9A). With the exception of two α-mannosidase families (GH38 and GH92), all GH families were found in equal or greater numbers in the C1 pangenome, including 9 families that were found only in that cluster (Fig. S9A). Mannose is among the main monosaccharides that constitute mucus glycoproteins, together with N-acetyl-α-galactosamine, N-acetyl-β-galactosamine, *N*-acetylglucosamine, fucose, and neuraminic acid, the latter two of which are found in the terminal residues of mucin glycoproteins (73).

10.1128/mBio.02852-18.9 FIG S9 Phenotypic analysis. (A) Percentage of glycoside hydrolases (GHs) in the pangenome detected using the Carbohydrate-Active enZYmes (CAZy) database. The red star indicates the results that differed using a criterion of a cutoff fold change value of greater than 1.5 between the clusters. In the box are the percentages of total glycoside hydrolases and polysaccharide lyases for both clusters. (B) Heat map of Biolog phenotypic microarrays measuring bacterial pH tolerance. Data are presented as growth curves of representatives for C1 (CMCP6; green circles) and C2 (ATCC 43382; blue triangles) in some carbon sources from a Biolog PM1 MicroPlate (Fig. 4C). Error bars represent standard deviations. (C) pH tolerance of representatives of C1 (CMCP6 and ATL9824) and C2 (ATCC 43382 and ORL1506) determined using PM10 phenotypic microarray plates. Experiments were conducted in duplicate. Download FIG S9, PDF file, 0.2 MB. Copyright © 2019 López-Pérez et al. 2019 López-Pérez et al. This content is distributed under the terms of the Creative Commons Attribution 4.0 International license.

The highest differences in abundances between the clusters corresponded to GH13 (α-amylase), GH23 (lysozyme with activity for several polysaccharides, including chitin), and GH109 (α-N-acetylgalactosaminidase) that might degrade the peptidoglycan of cell walls and mucus (74) (Fig. S9A). In order to experimentally determine the potential differences between C1 and C2 in carbohydrate utilization, we analyzed the growth of a couple of strains from each cluster (CMCP6 and ATL9824 [C1] and ATCC 43382 and ORL1506 [C2]) in a diverse range of carbon sources. We tested their ability to metabolize a variety of carbohydrates and other carbon sources using Biolog phenotypic microarray PM1 (Fig. 4C). Our analysis indicated that C1 is capable of utilizing a much larger range of carbon sources, in agreement with the genomic data of the pangenome (Fig. 4C; see also Fig. S9B). While members of the C1 grew better than the C2 representatives in 70 carbon sources, C2 outgrew C1 in only 14 (Fig. 4C). Specifically, C1 had at least twice the final optical density (OD) of C2 in d-mannitol, B-methyl-d-glucoside, a-d-lactose, and d-malic acid (Fig. 4C). Interestingly, the C2 ATCC 43382 strain was capable of utilizing l-fucose, a major component of the terminal glycans found in mucins, as a sole carbon source, whereas C1 representatives did not grow in the presence of l-fucose as a sole carbon source (Fig. S9B). Genome analysis results confirmed the phenotypic assay results, since ATCC 43382 contains genes encoding l-fucose permease in its genome, while CMCP6 does not. It has been demonstrated that uptake and utilization of l-fucose by Campylobacter jejuni, a prevalent gastrointestinal pathogen in humans, provided a distinct competitive advantage in its pathogenesis (75).

### Adaptations to eel mucosa colonization.

**FIG 5:**
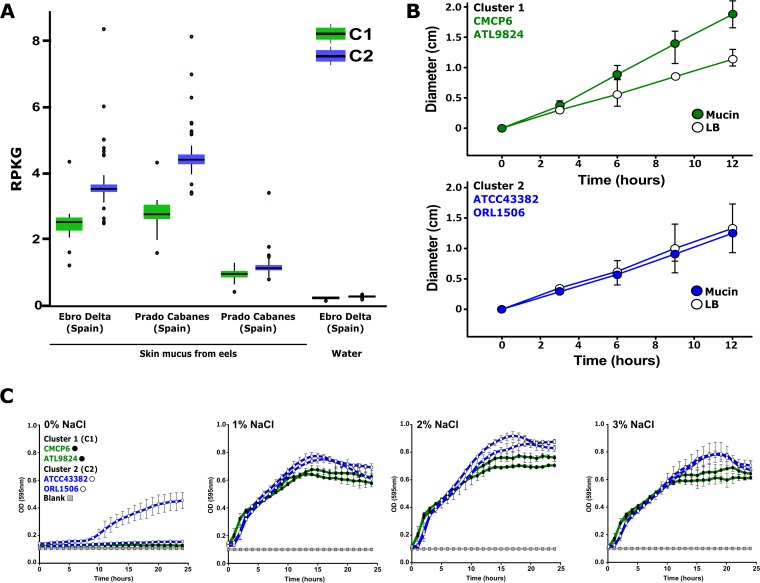
Adaptations of V. vulnificus strains to colonization of eel mucosa. (A) Relative abundances of members of the C1 and C2 based on metagenomic recruitment in eel skin mucus and seawater metagenome samples. Data are expressed as RPKG (reads recruited per kilobase of genome per gigabase of metagenome). (B) Motility assays of representatives of C1 (CMCP6 and ATL9824; green) and C2 (ATCC 43382 and ORL1506; blue) minimal media supplemented with mucin and LB at RT. Graphics represent the average diameter of motility zone of three replicates and error bars the standard deviation at 0 h, 3 h, 6 h, 9 h, and 12 h. (C) Growth of representatives of C1 (CMCP6 and ATL9824; green lines and white dots) and C2 (ATCC 43382 and ORL1506; blue lines and black dots) at different salinities (0% to 3% NaCl). Error bars represent standard deviations of results from three replicates.

Our analyses (phenotype and genomic evolution) and the distribution of the isolates indicate that the two main clusters of V. vulnificus have different lifestyles. Thus, we speculated that these phylogenetically distant clusters occupy different niches, possibly differing in their natural hosts or habitat. This physical isolation would result in distinct evolutionary pressures that would decrease the probability of encounter and recombination, ultimately leading to genetic isolation and the generation of distinct ecotypes. To test our hypothesis, we analyzed the abundance of both clusters through recruitment in the metagenome of the eel skin mucus and seawater (76). Even though all the sequenced strains isolated from healthy and diseased eels from worldwide locations belonged to C2 (Fig. 1), metagenomic recruitment showed that both clusters were present in the eel mucus. However, C2 was consistently found at greater frequencies than C1 (Fig. 5A) further supporting a scenario where it preferentially lives as a commensal of marine organisms. None of the clusters were detected in seawater metagenomes (Fig. 5A). V. vulnificus genomes do not generate a large number of reads from marine metagenomes, and most analysis of the possible host microbiota derived from 16S rRNA has shown that detection at the cluster level is impossible. It would be revealing to investigate the presence of members of the different clusters in other V. vulnificus hosts in future studies.

The consistent enrichment of C2 in the eel mucus metagenome prompted us to investigate the physiological adaptations that might lead to these differences. First, we investigated the motility response of C1 (strains CMCP6 and ATL9824) and C2 (strains ATCC 43382 and ORL1506) in the presence of mucin. We compared their motility characteristics in soft-agar plates containing M9 minimal media supplemented with 0.1% mucin or Luria-Bertani (LB) (Fig. 5B). We measured their motility zones at different time points for a period of 12 h. Strains from C1 exhibited greater motility in mucin than in LB media on average, while no significant differences between the two conditions were found for C2 (Fig. 5B). Therefore, C1 appears to react to some mucin components, increasing its motility in their presence. This has been previously identified in pathogenic strains of V. cholerae and other vibrios (77, 78). Our results are consistent with the differences in the pangenome for “motility and chemotaxis” in the SEED classification between the clusters and support the idea of a bloomer lifestyle for C1, chemotactic with respect to novel carbon sources, and of a commensal lifestyle for C2, adapted to live in a rich environment. The physicochemical parameters of the environment within the eel mucus differ from those of the surrounding environment. There are pH fluctuations, and the mean osmolarity of the eel mucus (∼1% NaCl) is lower than that of seawater (76). Thus, we examined the response of the two clusters to different pH-related conditions by testing their growth on Biolog phenotypic microarray PM10 (Fig. S9B). We found no major differences among the four strains from the two clusters that we analyzed under these conditions (Fig. S9C). We also tested growth under conditions of increased salinity (0% to 3% NaCl) (Fig. 5C). Interestingly, there were differences in growth on LB over the entire range of salinities (including the average mucus salinity level [1%]), where the C2 strains showed better growth than the representatives from C1 (Fig. 5C), which could be another important factor that explains the predominance of C2 in the metagenomes of the eel mucus.

## DISCUSSION

In order to emerge as a human pathogen, a bacterium must acquire numerous novel properties such as resistance to antimicrobials, avoidance of host immune defenses, or the ability to effectively colonize specific host tissues (66, 79). The acquisition and evolution of some of these pathogenic determinants are the results of the interaction of the bacterium with its natural habitat. These interactions prompt the selection of certain traits that increase its fitness in that ecological setting and also play a role in the context of the human host (26, 27). In this study, we investigated the population structure and genomic evolution of the marine pathogen V. vulnificus in order to understand the drivers that led to its emergence and cluster divergence.

The combined results of the different analyses in this study suggest that the population of V. vulnificus is made up of four distinct clusters. Although the ANI values within the different clusters were >97%, the divergence between the two largest clusters, C1 and C2, indicates that they are widely divergent lineages that are on the borderline of qualifying as different species. We speculate that the acquisition of different ecological determinants allowed the development of diverse lifestyles within the same environment, which has led to higher divergence. Interestingly, despite the genomic and ecological divergence of C1 and C2, the exchange of MGEs appears to happen frequently and indiscriminately, even between different species, as shown by the detection of identical elements in distant relatives (80).

**FIG 6:**
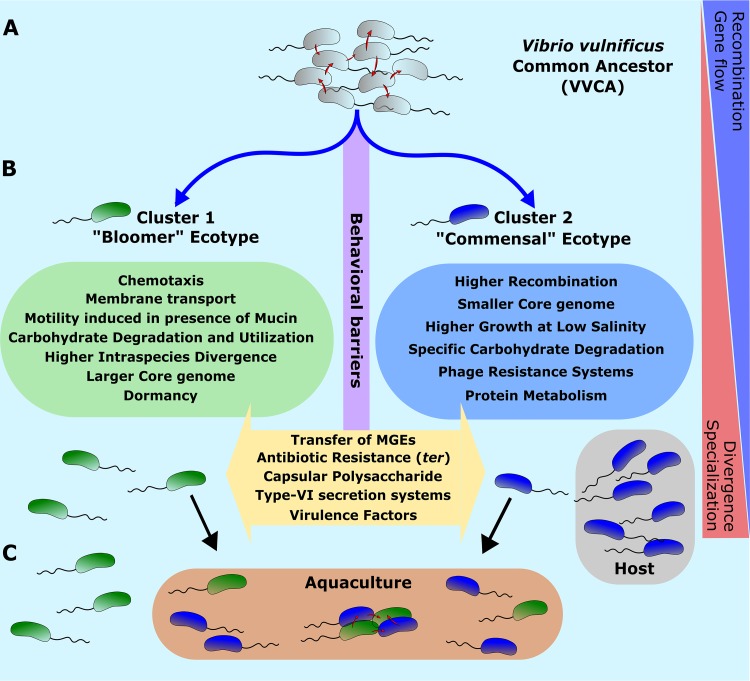
Evolutionary model of cluster divergence in V. vulnificus. (A) VVCA. Clonal lineages start diverging from the V. vulnificus common ancestor (VVCA). (B) Divergence. The acquisition of different ecological determinants allowed the development of diverse lifestyles within the same environment, which has led to a higher divergence. This divergence led to a recombination and gene flow decrease, although frequent exchange of mobile genetic elements is found within the species and with other species. (C) Convergence. With the advent of aquaculture, we have created an artificial environment that has led to colocalization of strains from the two major clusters.

It appears that C2 members have a competitive advantage for colonization and growth in different hosts following a commensal lifestyle, which might be a reason explaining why the greatest number of isolates has been obtained from this cluster (Fig. 6B). This specialization model could explain the smaller core genome and lower divergence of the members of C2 and their prevalence in nutrient-rich environments such as mucous surfaces. Our results indicate that C1 is a bloomer that grows when the conditions are favorable due to the high potential to degrade carbohydrates, a greater proportion of secretion systems, or a higher abundance of genes related to “dormancy and sporulation,” which support a “feast to famine” lifestyle allowing bacterial cells to endure long periods of unfavorable environmental conditions (68, 69) (Fig. 6B). Their ability to use a greater pool of nutrients and to tolerate a larger range of stressors in the environment likely provides an advantage to V. vulnificus C1 in coping with the rapid and drastic ecological transition under the unfavorable conditions of the oligotrophic aquatic environments.

Our scenario proposes that strains from the two clusters occupy different niches that lead over time to a greater divergence of the two ecotypes. We contend that these continuing divergences would likely eventually lead to speciation of the two clusters (Fig. 6B). Interestingly, it appears that with the advent of aquaculture we have created an artificial environment where the two clusters can be isolated in sympatry; while C2 is commensal of the eel in aquaculture, the confinement of the fish, together with the organic matter that is added and their depositions, might produce the ideal environment for a continuous bloom of members of C1 (Fig. 6C). Furthermore, this newly created artificial environment increases the possibility of contact between the clusters, thus maximizing the probability of transfer of genetic material and of recombination. This could entail a risk of emergence of novel clusters with potentially devastating consequences for both aquaculture and human health.

Overall, our results shed light on some of the underlying genomic properties associated with the emergence of pathogenic V. vulnificus. We consider that our findings could provide information relevant to the pursuit of strategies to prevent and foresee the potential emergence of strains with importance to both human health and aquaculture. Finally, our evolutionary model and genomic approaches are broadly applicable to other pathogenic vibrios and facultative bacterial pathogens.

## MATERIALS AND METHODS

### Bacterial isolates and population structure.

We downloaded all the genomes present in the NCBI belonging to the V. vulnificus species. Genomic features, cluster affiliations, and origins of the 113 V. vulnificus strains used are listed in Table S1 in the supplemental material. Reciprocal BLASTN and TBLASTX searches were carried out between the genomes, leading to the identification of regions of similarity, insertions, and rearrangements. The values representing ANI between strains were calculated using JSpecies software package v1.2.1 and default parameters (81).

### SNPs, population structure, and recombination analyses.

The population structure of V. vulnificus was reconstructed using STRUCTURE (82). The number of hypothetical ancestral populations (*K*) was estimated to be equal to *K* = 4. The optimum *K* value was evaluated by the Δ*K* method (83) using independent runs for a number of populations *K* ranging from 2 to 10. The Harvest Suite, a software package which includes tools such as Parsnp and Gingr, was used to perform the core alignment and to obtain the SNPs between strains (84). Indels and SNPs between small regions of the genome such as genomic islands were identified using the nucmer program in the MUMmer3+ package (85). ClonalFrameML (34) was also used with default parameters to take into account recombination events and to calculate the R/theta ratios (relative rates of recombination and mutation). GenBank files from all the strains downloaded from the NCBI were converted to GFF files. These files were used to estimate the pangenome using the Roary pipeline with a 70% identity cutoff value (86).

### Phylogenomic reconstructions.

The core genome SNP analyses for all the strains were performed using the KSNP v3.0 program (87) with the optimum kmer size of 19, which was determined by Kchooser. Maximum likelihood trees for the two chromosomes were generated individually using RAxML (version 7.2.6) (88), and the core alignment was obtained with Parsnp software. Then, the file was edited using iTool v3 software (89).

### Evolutionary rate.

To calculate the nonsynonymous (d*N*) and synonymous (d*S*) substitutions for an ortholog in a pair of V. vulnificus strains, we used the orthologr package (90). Briefly, this package identifies orthologous gene pairs by choosing the best reciprocal hit using BLASTp and performs codon alignments of the orthologous gene pairs using PAL2NAL (91). Finally, GESTIMATOR (92) computes the d*N*/d*S* values of the codon alignments. A low ratio (d*N*/d*S* < 1) indicates purifying selection, whereas a high ratio (d*N*/d*S* > 1) is a clear signal of diversifying selection.

### Functional classification.

Putative functionality, the presence of virulence factors, and the presence of antibiotic resistance factors encoded in the genomes were inferred by comparing all the proteins against the SEED subsystem database (64), virulence factor database (VFDB) (38), and MEGARes database (39), respectively. Proteins were compared using the different databases and DIAMOND (93) (blastp option, top hit, ≥50% identity, ≥50% alignment length, E value of <10 − 5). Using dbSCAN (94), we analyzed the presence of glycoside hydrolases, comparing all the proteins against the Carbohydrate-Active enZYmes (CAZy) database (72).

### Strains and culture conditions.

The experimental analyses utilized Vibrio vulnificus isolates CMCP6 and ATL9824 as representatives of C1 (laboratory collection) and ATCC 43382 (American Type Culture Collection, Rockville, MD) and ORL1506 (Paul Gulig) as representatives of C2. All strains were routinely grown in Luria-Bertani (LB) broth or agar plates containing 2% (wt/vol) NaCl for 16 h aerobically at 37°C, unless otherwise specified.

### Biolog phenotypic microarrays.

Differences in carbon utilization and pH tolerance were assessed using the Biolog microbial identification system (Biolog, Hayward, CA). Phenotypic MicroArray 1 (PM1) and PM10 analyses were carried out in duplicate following the manufacturer’s instructions. Briefly, colonies from agar plates were suspended in 1× IF-0a (PM1) or IF-10 (PM10) inoculation media supplemented with NaCl for a final concentration of 1% (wt/vol), as recommended by the manufacturer, and a 1:5 dilution (PM1) or 1:200 dilution (PM10) of this suspension was prepared to obtain an absorbance of 0.07 at 600 nm. Aliquots (100 µl) of the final cell suspension were added to each well. The plates were incubated under aerobic conditions at 37°C for 48 h with shaking. The optical density (OD) was measured at 595 nm every hour for 48 h using a Tecan Sunrise microplate reader, and the results were evaluated using Magellan plate reader software. Growth curves were plotted using GraphPad Prism V7, and area under the curve (AuC) values were calculated. Data were normalized by taking the ratio of the AuC of the respective carbon sources or pH conditions to that of the negative control. Normalized data were used to plot a heat map to compare the strains.

### Metagenomic read recruitments.

C1 and C2 members were used to recruit reads from eel mucus and seawater metagenomics data sets (76) using BLASTN and a cutoff value of 99% nucleotide identity over a minimum alignment length of 50 nucleotides. Hits obtained were used to compute the RPKG (reads recruited per kilobase of genome per gigabase of metagenome) values that provide normalized numbers that are comparable across various metagenomes.

### Growth curves at different salinities.

For the growth curve analysis, the overnight cultures of V. vulnificus strains were centrifuged to obtain a pellet, washed with LB, and resuspended in LB media containing no salt. Dilutions (1:100) of the cell suspensions were prepared in LB media containing final concentrations of 0%, 1%, 2%, and 3% (wt/vol) NaCl. Aliquots (200 µl) of each suspension were added to a 96-well microtiter plate. The experiment was performed in triplicate, with three independent biological replicates. Growth curves were plotted using GraphPad Prism V7.

### Motility assays.

Assessment of motility was performed using soft-agar motility plates containing 0.3% (wt/vol) agar and either LB or M9 minimal media supplemented with 0.1% (wt/vol) mucin from porcine stomach (Sigma). Single colonies were stabbed in the center of the ∼10-cm-diameter soft-agar plates using a sterile inoculating loop. Plates were incubated at room temperature (RT). The diameter of the motility zone was measured at the time points indicated in the *x* axis. Experiments were conducted in triplicate. Graphs were plotted using GraphPad Prism software, V7. The motility characteristics of the two strains on the different media were compared using Student's *t* test (*, *P* < 0.05; **, *P* < 0.005).
