# Supplementary figures and images for: Evolutionary Model of Cluster Divergence of the Emergent Marine Pathogen Vibrio vulnificus: From Genotype to Ecotype
Source: mBio. 2019 Feb 19;10(1):e02852-18. doi: 10.1128/mBio.02852-18 (PMC6381281; doi:10.1128/mBio.02852-18)

# V. vulnificus Cluster-1

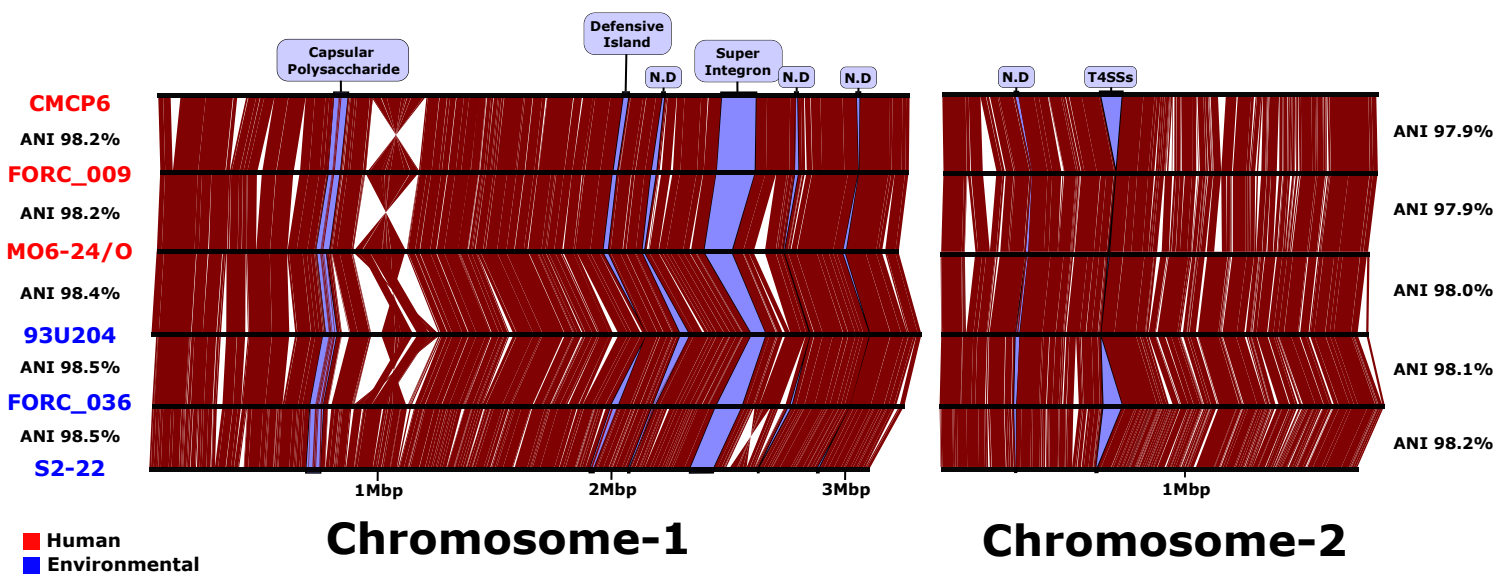

# V. vulnificus Cluster-2

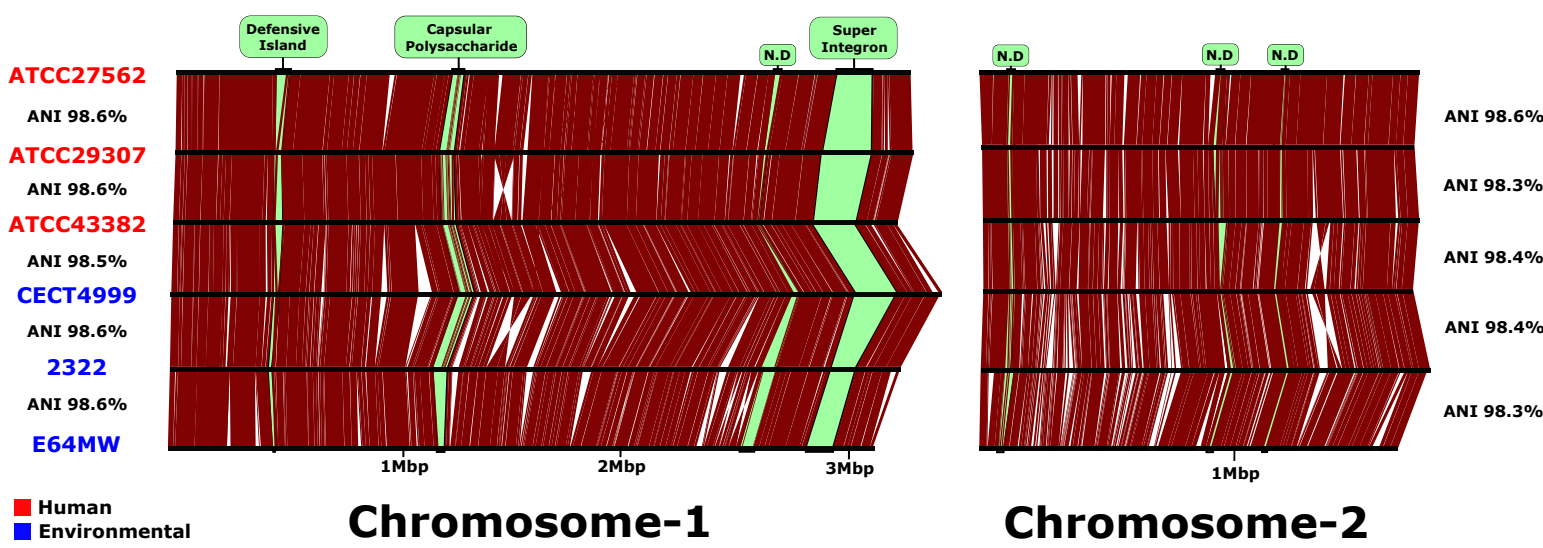

Supplement: FIG S2 [file mBio.02852-18-sf002.pdf]

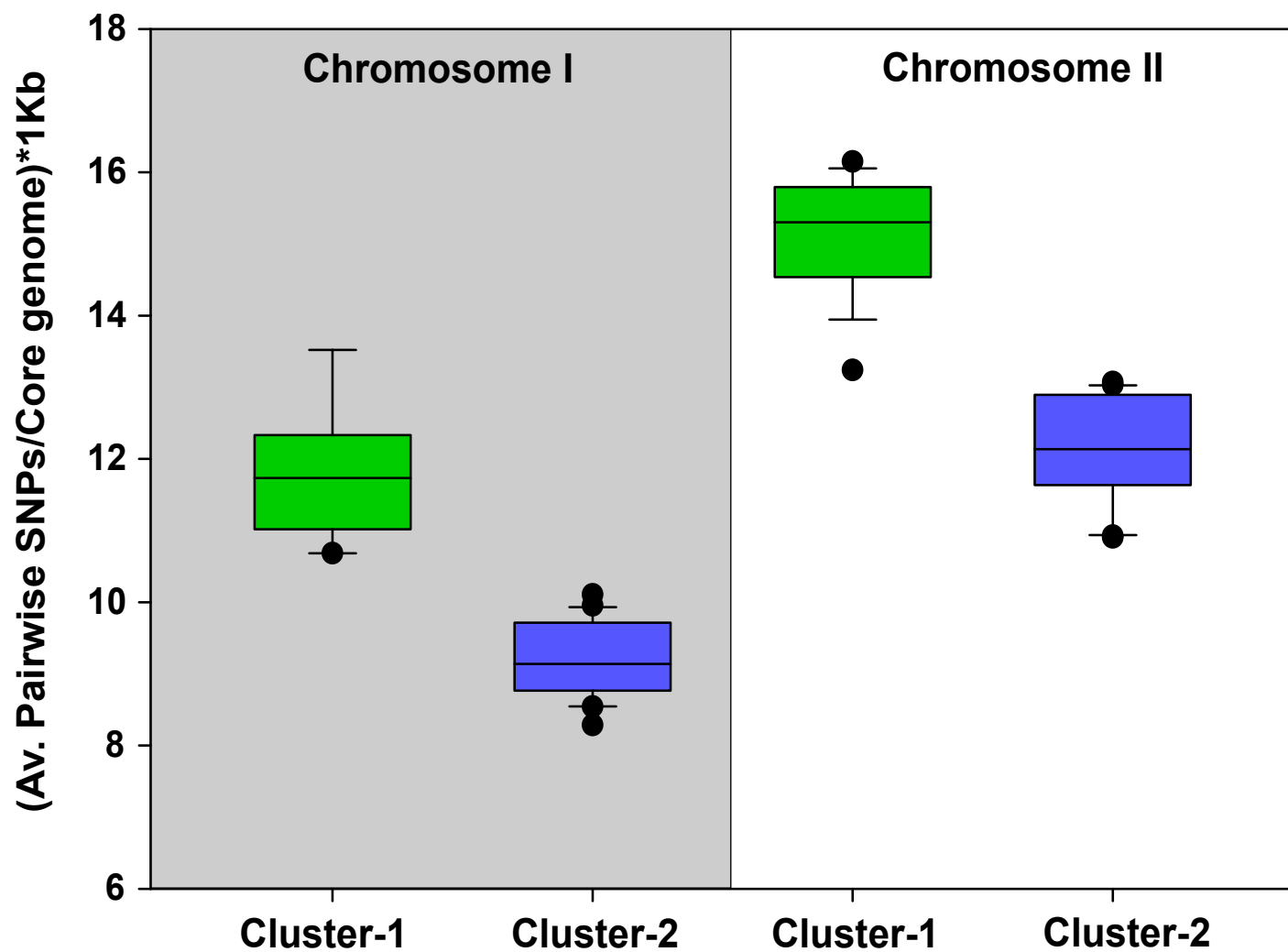

Supplement: FIG S3 [file mBio.02852-18-sf003.pdf]

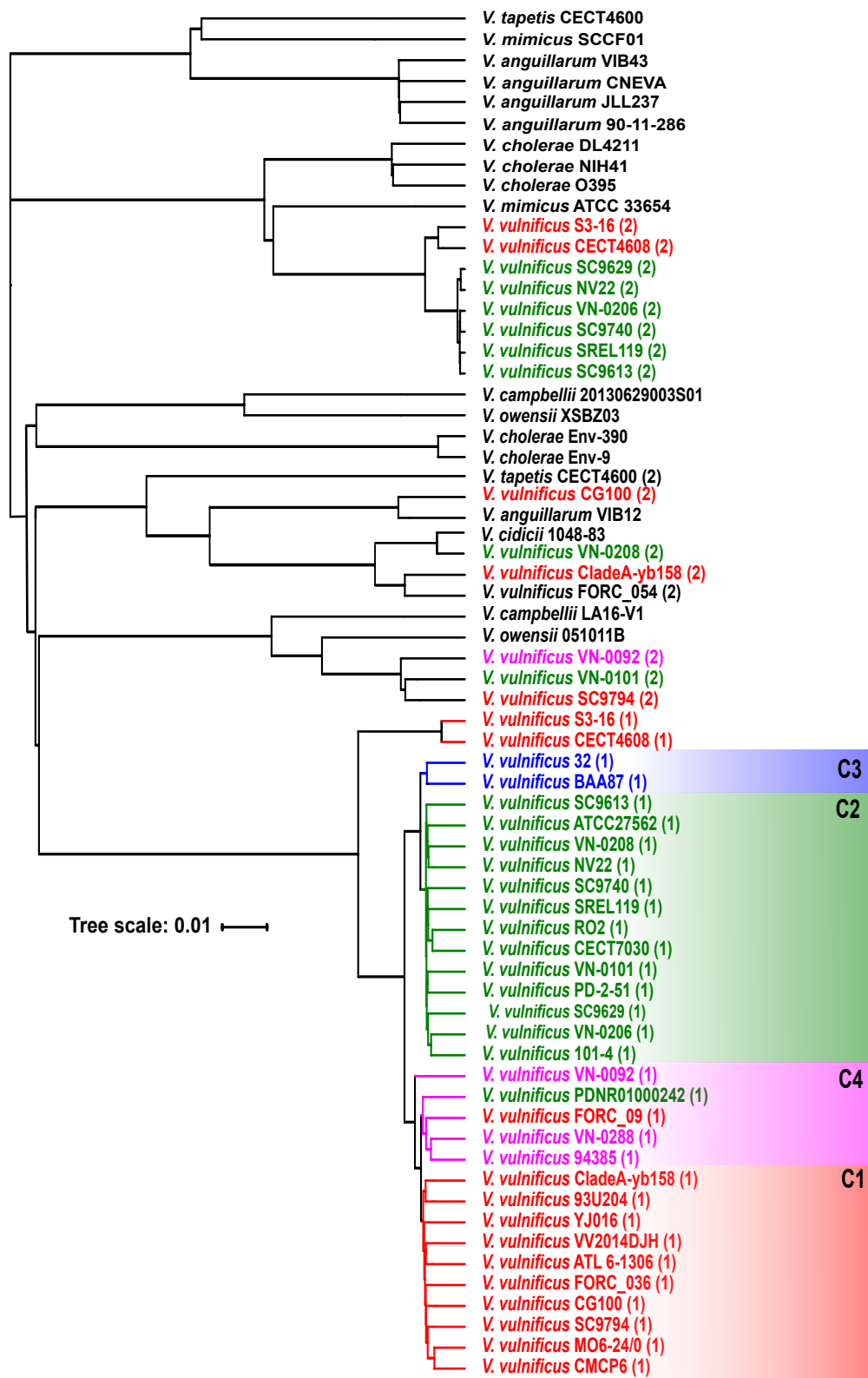

Supplement: FIG S5 [file mBio.02852-18-sf005.pdf]

**A**

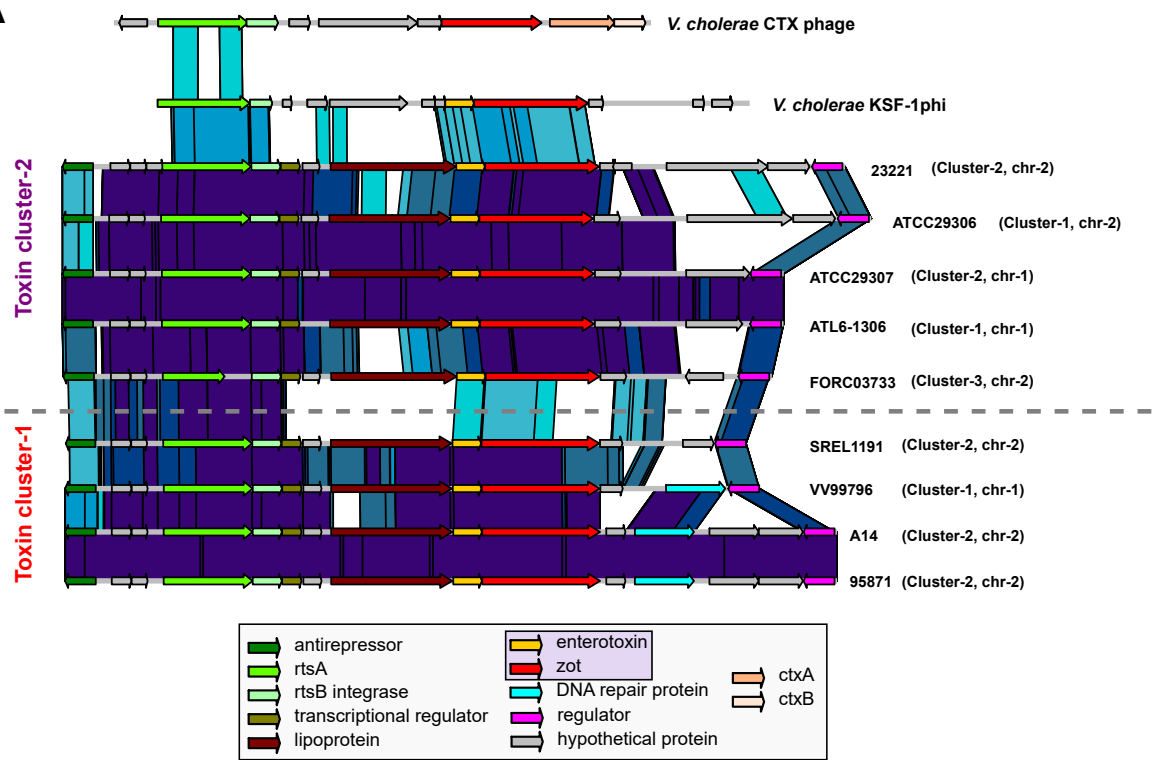

**B**

### Zonular occludens toxin Phylogenetic tree

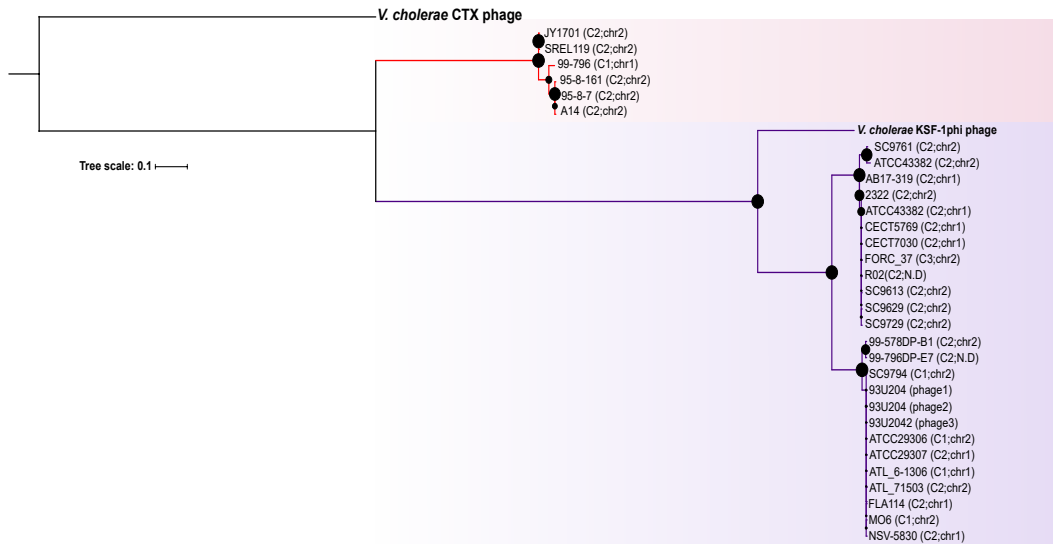

**C**

### Enterotoxin Phylogenetic tree

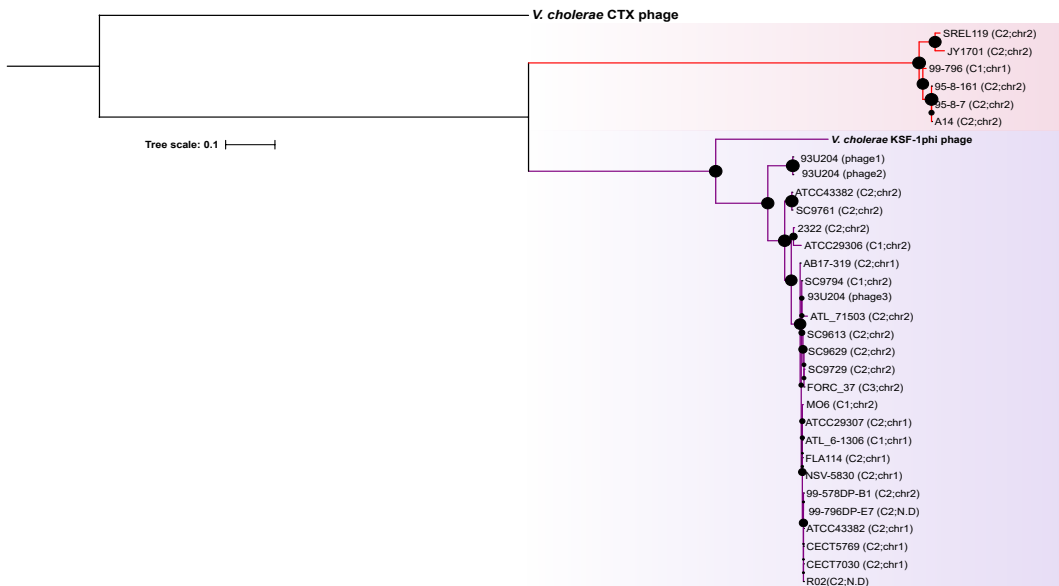

Supplement: FIG S7 [file mBio.02852-18-sf007.pdf]

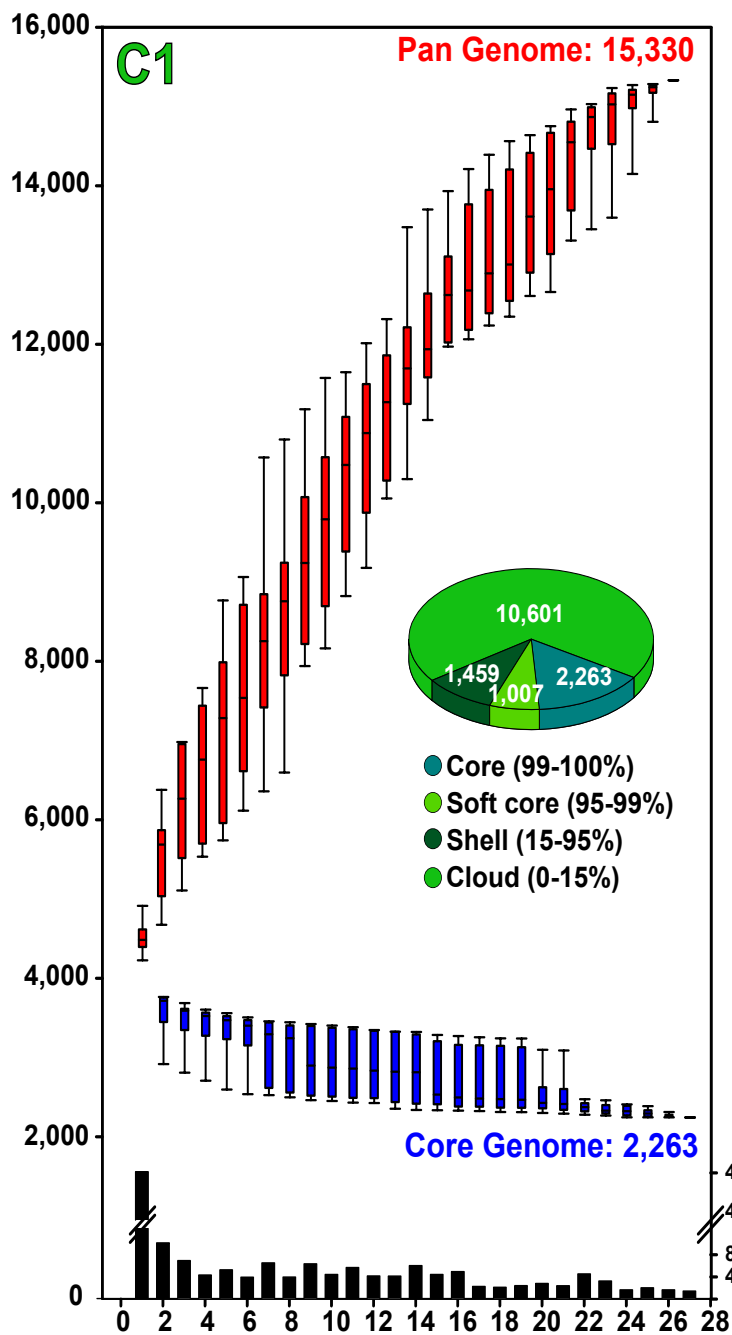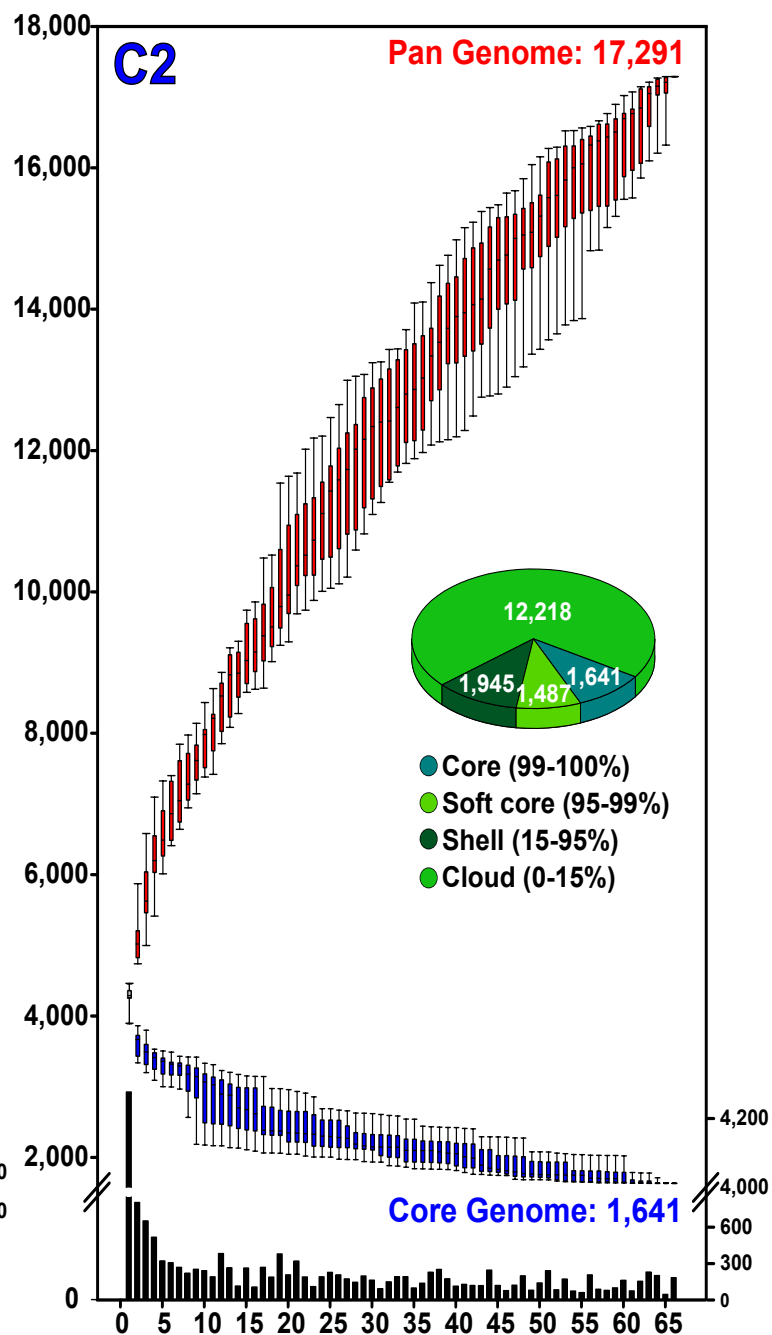

Supplement: FIG S8 [file mBio.02852-18-sf008.pdf]

A

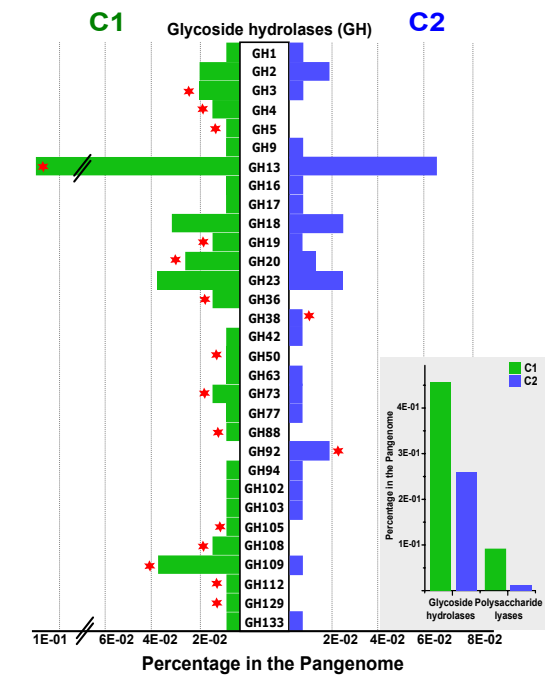

B

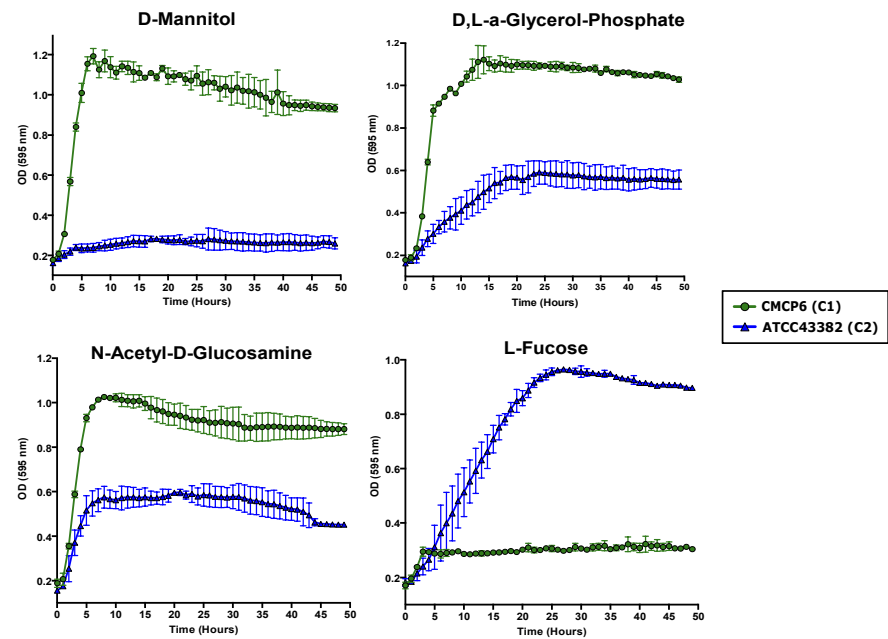

C

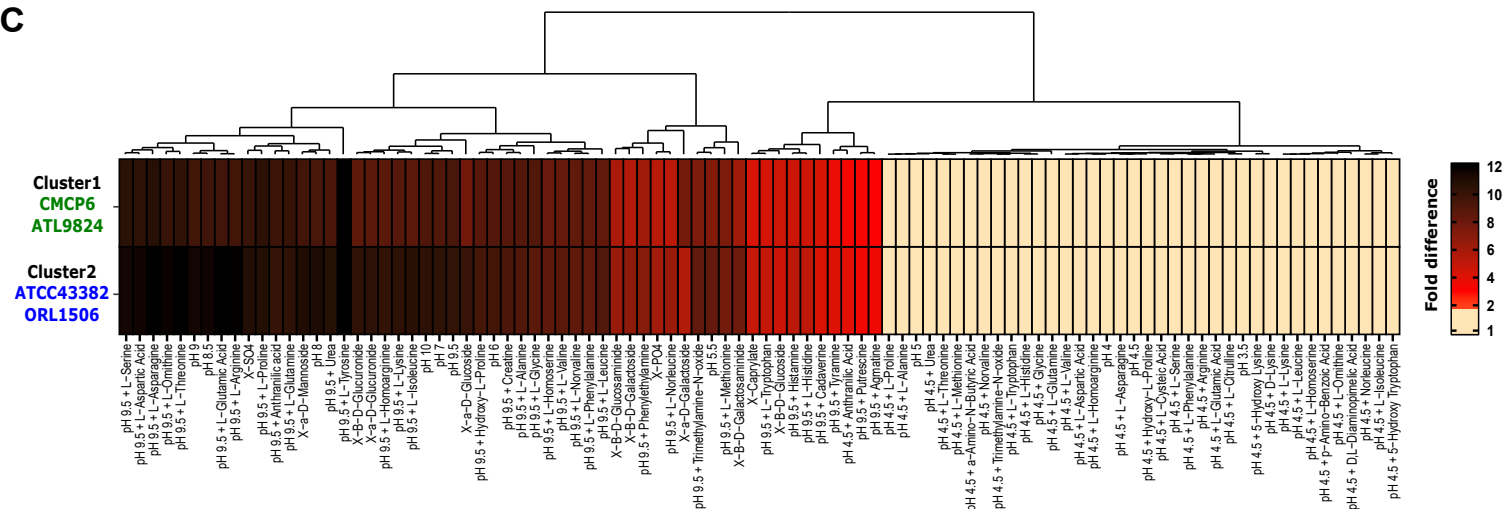

Supplement: FIG S9 [file mBio.02852-18-sf009.pdf]
