# Supplementary material for: Evolutionary Model of Cluster Divergence of the Emergent Marine Pathogen Vibrio vulnificus: From Genotype to Ecotype
Source: mBio. 2019 Feb 19;10(1):e02852-18. doi: 10.1128/mBio.02852-18 (PMC6381281; doi:10.1128/mBio.02852-18)

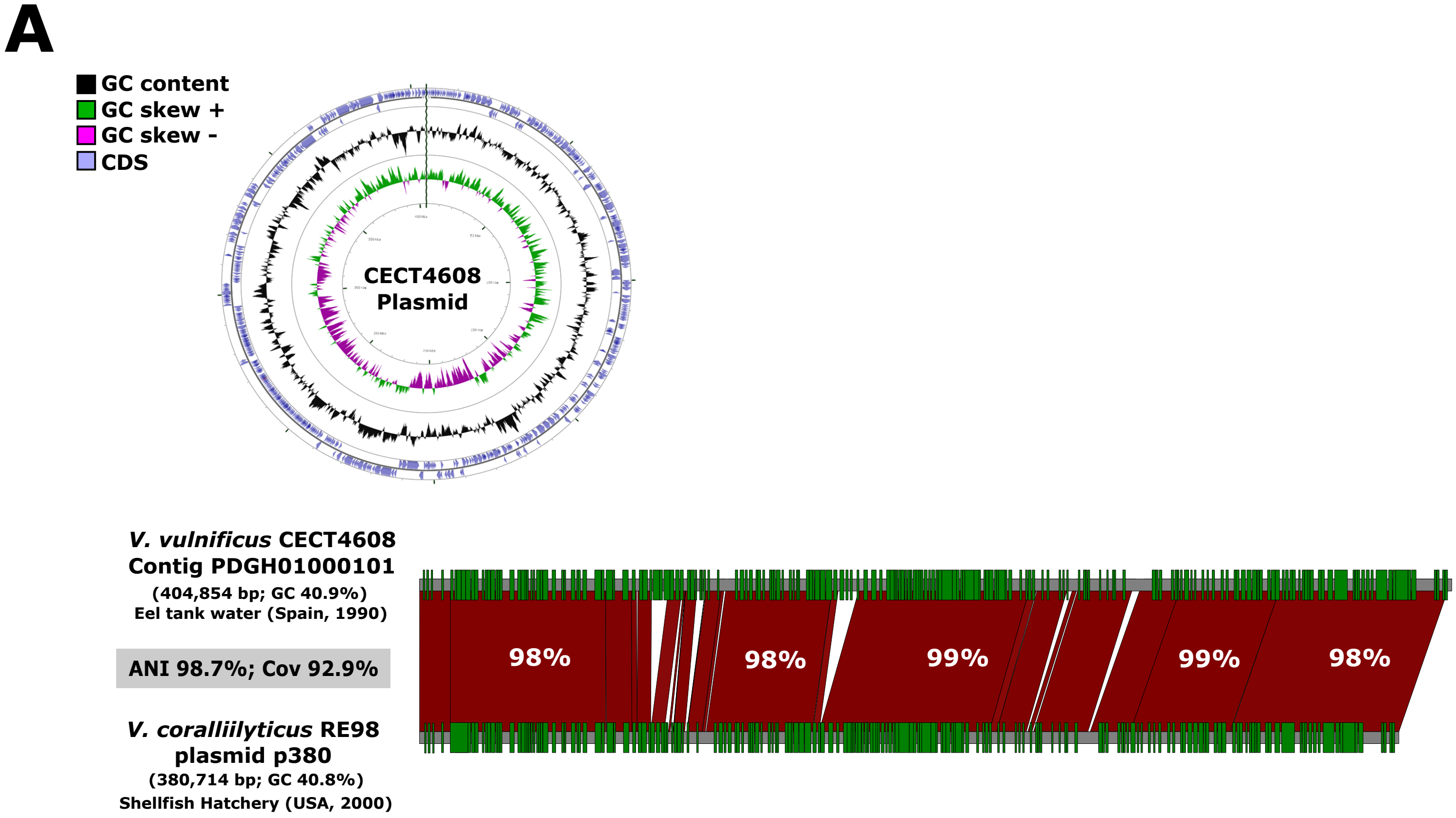

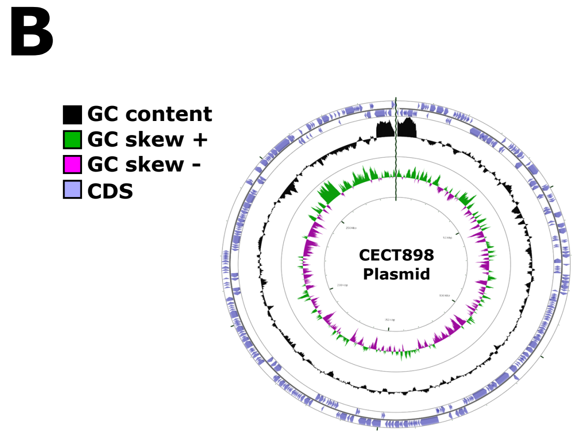

	Strain	Host	Isolation area	Year
Vibrio vulnificus	CECT898	Diseased eel	Japan	1979
Vibrio harveyi	ZJ0603	Orange-spotted grouper	China	2008
Vibrio cholerae	116-14	Seepage water sample	India	2015
Vibrio parahaemolyticus	VPS92	Shrimp (supermarket)	China	2015
Klebsiella pneumoniae	CGMHLK78	Homo sapiens	Taiwan	2013

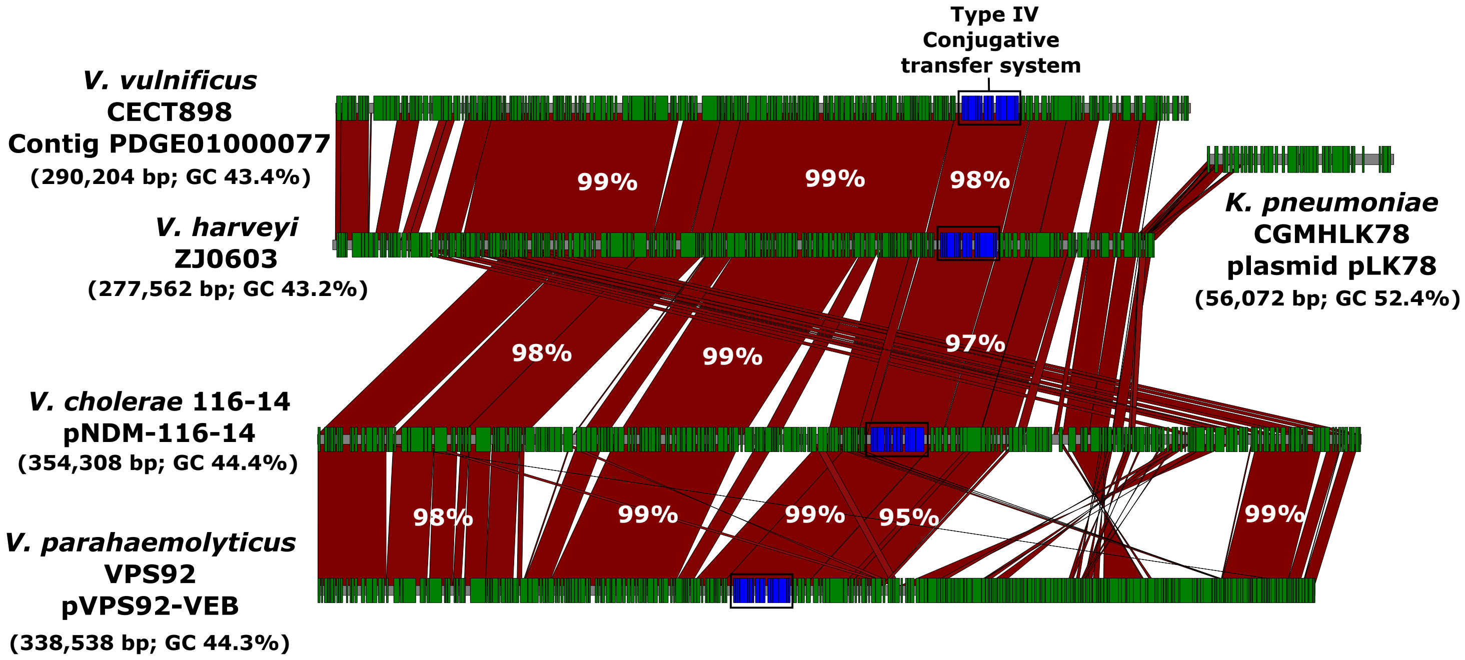

Supplement: FIG S6 [file mBio.02852-18-sf006.pdf]
