# Supplementary material for: Evolutionary Model of Cluster Divergence of the Emergent Marine Pathogen Vibrio vulnificus: From Genotype to Ecotype
Source: mBio. 2019 Feb 19;10(1):e02852-18. doi: 10.1128/mBio.02852-18 (PMC6381281; doi:10.1128/mBio.02852-18)
Supplement: FIG S1 [file mBio.02852-18-sf001.pdf]

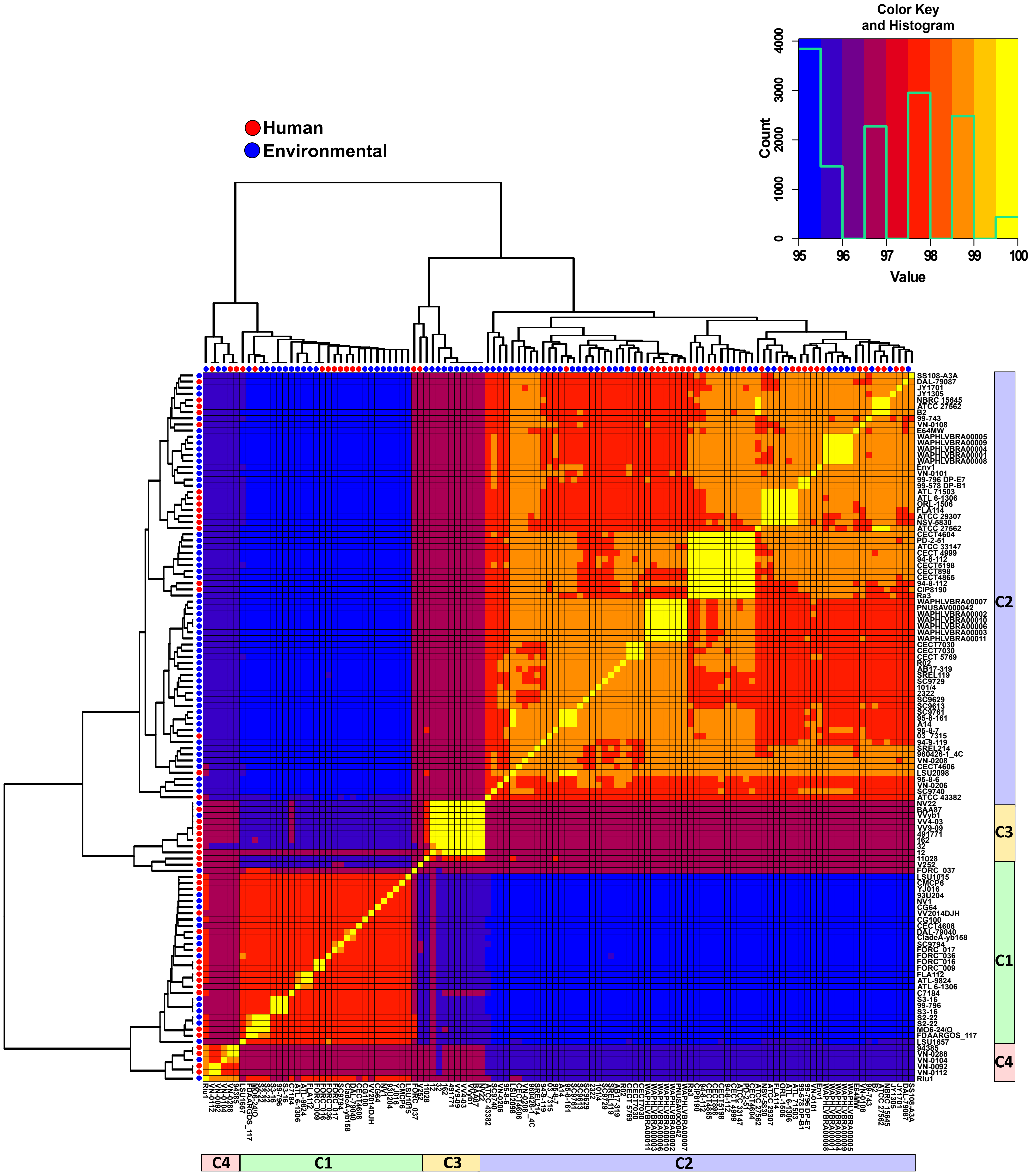
Color Key
and Histogram
Count
Value
95
96
97
98
99
100
0
1000
2000
3000
4000
Human
Environmental
C1
C2
C3
C4
