# Supplementary material for: Evolutionary Model of Cluster Divergence of the Emergent Marine Pathogen Vibrio vulnificus: From Genotype to Ecotype
Source: mBio. 2019 Feb 19;10(1):e02852-18. doi: 10.1128/mBio.02852-18 (PMC6381281; doi:10.1128/mBio.02852-18)
Supplement: FIG S4 [file mBio.02852-18-sf004.pdf]

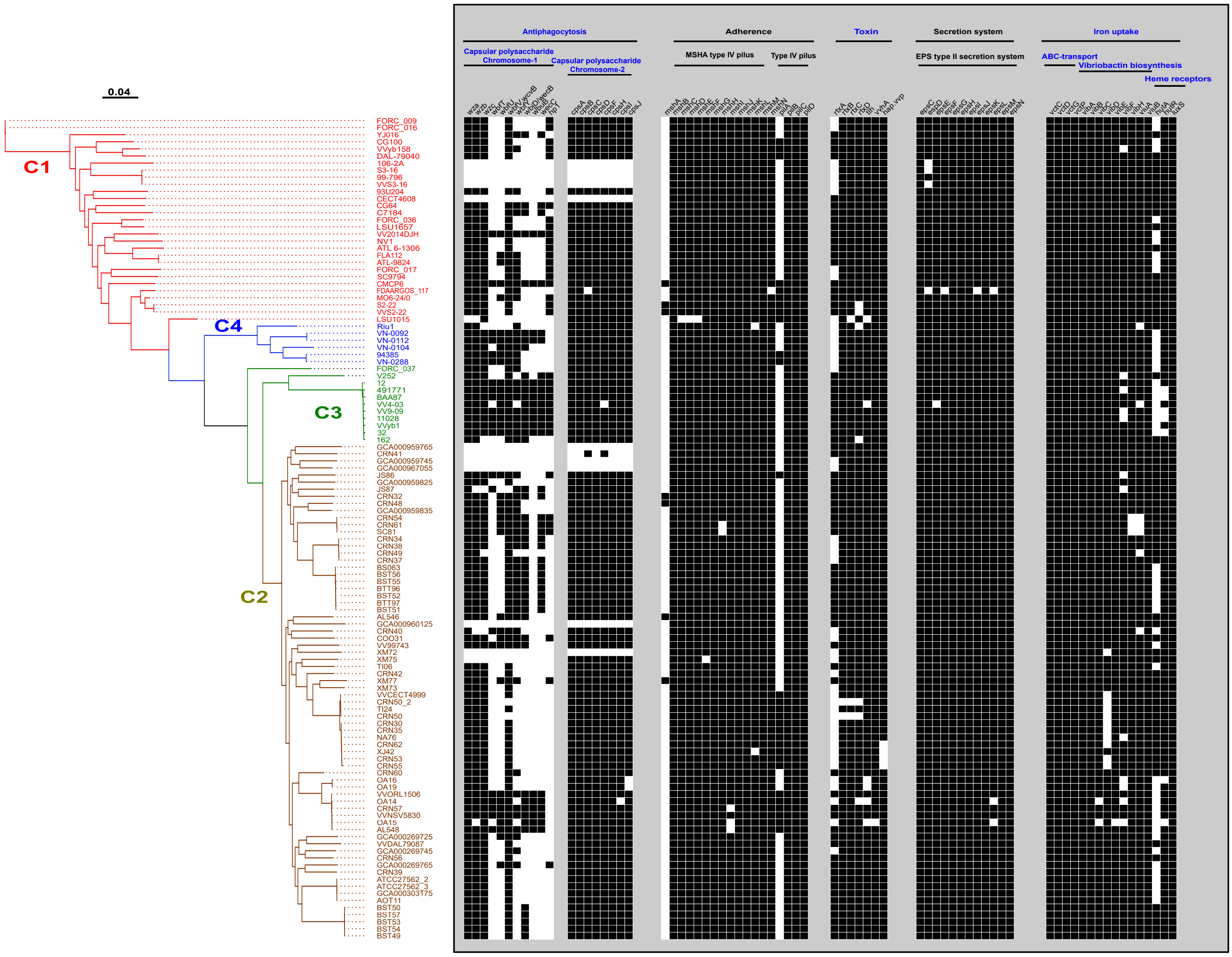

0.04
C1
C4
C3
C2
Antiphagocytosis
Capsular polysaccharide Chromosome-1
Capsular polysaccharide Chromosome-2
Adherence
MSHA type IV pilus
Type IV pilus
Toxin
Secretion system
EPS type II secretion system
Iron uptake
ABC-transport
Vibriobactin biosynthesis
Heme receptors
FORC_009
FORC_016
YJ016
CG100
VVyb158
DAL-79040
106-2A
S3-16
99-796
VVS3-16
93U204
CECT4608
CG64
C7184
FORC_036
LSU1657
VV2014DJH
NV1
ATL 6-1306
FLA112
ATL-9824
FORC_017
SC9794
CMCP6
FDAARGOS_117
MO6-24/O
S2-22
VVS2-22
LSU1015
Riu1
VN-0092
VN-0112
VN-0104
94385
VN-0288
FORC_037
V252
12
491771
BAA87
VV4-03
VV9-09
11028
VVyb1
32
162
GCA000959765
CRN41
GCA000959745
GCA000967055
JS86
GCA000959825
JS87
CRN32
CRN48
GCA000959835
CRN54
CRN61
SC81
CRN34
CRN38
CRN49
CRN37
BS063
BST56
BST55
BTT96
BST52
BTT97
BST51
AL546
GCA000960125
CRN40
CO031
VV99743
XM72
XM75
TI06
CRN42
XM77
XM73
VVCECT4999
CRN50_2
TI24
CRN50
CRN30
CRN35
NA76
CRN62
XJ42
CRN53
CRN55
CRN60
OA16
OA19
VVORL1506
OA14
CRN57
VVNSV5830
OA15
AL548
GCA000269725
VVDAL79087
GCA000269745
CRN56
GCA000269765
CRN39
ATCC27562_2
ATCC27562_3
GCA000303175
AOT11
BST50
BST57
BST53
BST54
BST49
